# A Reversible Phase Transition of 2D Coordination Layers by B–H∙∙∙Cu(II) Interactions in a Coordination Polymer

**DOI:** 10.3390/molecules24173204

**Published:** 2019-09-03

**Authors:** Lei Gan, Pol G. Fonquernie, Mark E. Light, Gantulga Norjmaa, Gregori Ujaque, Duane Choquesillo-Lazarte, Julio Fraile, Francesc Teixidor, Clara Viñas, José G. Planas

**Affiliations:** 1 Institut de Ciència de Materials de Barcelona (ICMAB-CSIC), 08193 Bellaterra, Spain; 2 Department of Chemistry, University of Southampton, Highfield, Southampton SO17 1BJ, UK; 3 Departament de Química, Universitat Autònoma de Barcelona, 08193 Cerdanyola del Valles, Catalonia, Spain; 4 Laboratorio de Estudios Cristalográficos, IACT, CSIC-Universidad de Granada, Av. de las Palmeras 4, E-18100 Armilla, Granada, Spain

**Keywords:** metal-organic frameworks, open metal sites, boron hydrides, carborane, reversible phase transition

## Abstract

Materials that combine flexibility and open metal sites are crucial for myriad applications. In this article, we report a 2D coordination polymer (CP) assembled from CuII ions and a flexible *meta*-carborane-based linker [Cu_2_(L1)_2_(Solv)_2_]•xSolv (1-DMA, 1-DMF, and 1-MeOH; L1: 1,7-di(4-carboxyphenyl)-1,7-dicarba-closo-dodecaborane). 1-DMF undergoes an unusual example of reversible phase transition on solvent treatment (i.e., MeOH and CH_2_Cl_2_). Solvent exchange, followed by thermal activation provided a new porous phase that exhibits an estimated Brunauer-Emmett-Teller (BET) surface area of 301 m^2^ g^−1^ and is capable of a CO_2_ uptake of 41 cm^3^ g^−1^. The transformation is reversible and 1-DMF is reformed on addition of DMF to the porous phase. We provide evidence for the reversible process being the result of the formation/cleavage of weak but attractive B–H∙∙∙Cu interactions by a combination of single-crystal (SCXRD), powder (PXRD) X-ray diffraction, Raman spectroscopy, and DFT calculations.

## 1. Introduction

Porous Coordination Polymers (CPs) or Metal-Organic Frameworks (MOFs) are a class of porous crystalline materials formed by the assembly of metal ions or metal clusters with different types of bridging organic linkers or ligands [1,2,3,4]. Open metal sites (OMS) in these materials, where Lewis base molecules can coordinate or interact, are known to play an important role in a variety of applications, ranging from catalysis [5,6,7] to molecules storage [8,9], separation [10,11,12], or sensing [13,14]. Such interactions often involve molecules including H_2_O, CO, CO_2_, H_2_, CH_4_, N_2_, or H_2_ among others and undergo dynamic processes that are difficult to probe directly by spectroscopic experiments such as infrared or Raman spectroscopy [15,16,17]. Among the MOFs that can easily generate OMS, Cu(II)-based paddlewheel MOFs have been extensively studied and exhibit structural diversity and high porosity [18,19,20]. Solvent molecules bound to the apical position of the Cu_2_-paddlewheel motif (*vide infra*) can be removed to generate unsaturated Cu(II) OMS. Among the various strategies for MOFs activation (removal of pore-filling and metal coordinated solvent), only thermal activation, which consists of applying heat energy and vacuum, has so far been able to dissociate coordinating solvent and generate OMS [15,21,22]. In a series of papers [23,24,25,26], N. C. Jeong and coworkers have nicely demonstrated that the Cl atoms of dichloro- or trichloro methane can weakly coordinate to the open metal sites of well-known Cu(II) MOFs such as HKUST-1 (Copper benzene-1,3,5-tricarboxylate) or Cu-MOF-2 (Copper 1,4-benzene-dicarboxylate). Such weak coordination of chloromethanes leads to what the authors termed a chemical route to active open metal sites by removing coordinating solvent. They proved the ability of chloromethanes to remove precoordinated solvent molecules from OMS, by in situ Raman spectroscopy, and therefore leading to MOF activation without the need for thermal activation. Such a mild activation in solution opens new avenues for potential applications of MOFs [24,27].

**Scheme 1:**
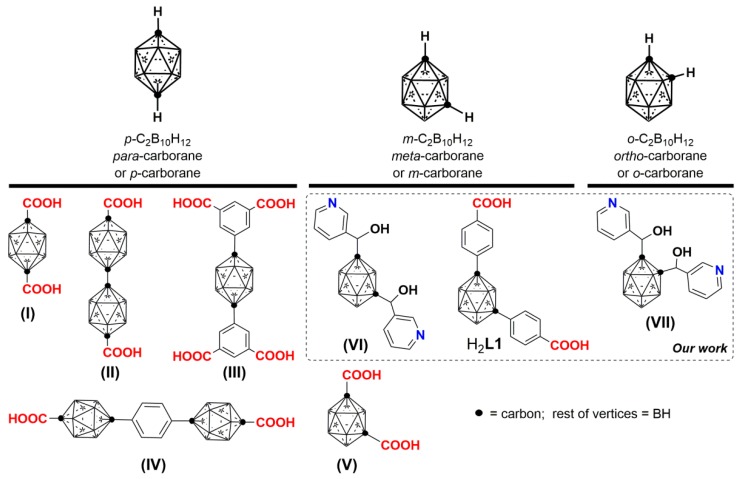
Graphical representation of the carborane isomers (*closo*-C_2_B_10_H_12_) and their derivatives used as linkers to form CPs.

Icosahedral boranes and carboranes ([B_12_H_12_]^2−^ and 1,n-C_2_B_10_H_12_ (n = 2,7 or 12); Scheme 1) are an interesting class of commercially available and exceptionally stable 3D-aromatic boron-rich clusters that possess material-favorable properties such as thermal and chemical stability and hydrophobicity [28,29,30]. The neutral carboranes are remarkably robust boron clusters with two carbon atoms and possess 26 electrons for 12 vertices. The delocalized electron density is not uniform through the cage, giving rise to extraordinary differences in the electronic effects of the cluster [31]. This unusual electronic structure is often highlighted by regarding carboranes as inorganic three-dimensional “aromatic” analogs of arenes [32]. Such properties make icosahedral carborane clusters valuable ligands for CPs or MOFs. For example, some of us have reported the synthesis and electronic and magnetic properties of purely inorganic CPs based on the monocarboxylic acid of *ortho*-carborane [33,34,35]. Mirkin and co-workers explored the use of di-, tri- and tetra-carboxylic acid derivatives of *para*-carborane (**I** to **IV** in Scheme 1) for CPs synthesis, providing a series of CPs exhibiting unprecedented stabilities with respect to thermal degradation, inherited from the carborane moiety [36,37,38,39,40,41,42]. Jin and co-workers also constructed CPs based on the dicarboxylic acid derivatives of *para*- but also of *meta*-carborane linkers (**V** in Scheme 1), and studied their adsorption and luminescence properties [43,44]. Dicarboxylic and tricarboxylic derivatives of the smaller carborane *closo*-1,10-C_2_B_8_H_10_ were also incorporated into porous CPs [38,45]. We have recently designed flexible carborane-based ligands for dynamic MOFs (**VI** and **VII** in Scheme 1) [46,47]. In addition to water stability [47], the spherical shape of the carborane moiety in these flexible linkers seems to have a noticeable influence in the dynamic behavior of the MOFs. Thus, we previously reported an unusual reversible 3D to 2D transformation of a Cobalt-based MOF, incorporating a carborane-based dipyridine ligand [48]. We argued that contrary to classical flat aromatic ligand-based MOFs, spherical-shape linkers such as carboranes, can access extensive conformational space by a combination of low-energy torsion of the substituents and by the spherical core of the ligand. Such spherical carborane-based ligands can facilitate multiple supramolecular contacts that are of a different nature to those found in conventional planar carbon-based ligands. These include B–H∙∙∙A (A = H [49,50,51], I [52], π [53,54]) interactions or B–H∙∙∙Metal [55,56,57,58,59,60,61,62] (agostic) interactions in molecular systems. The latter type of interaction is well documented in icosahedral cages and is considered a preliminary step in many B–H activation reactions and in hydrogenation or hydroboration [62,63,64]. The hydridic nature of the H atoms in boranes and carboranes make these BH moieties suitable for metal coordination [65,66]. During the preparation of this manuscript, it has been reported a first example of a B–H∙∙∙Cu(II)-based MOF from [B_12_H_12_]^2−^ [67].

Here we report the synthesis of a new family of Cu-paddlewheel-based 2D coordination polymers incorporating a dicarboxylate *m*-carborane ligand [Cu_2_(L1)_2_(Solv)_2_]•xSolv (L1: 1,7-di(4-carboxyphenyl)-1,7-dicarba-*closo*-dodecaborane; Scheme 1) and an unprecedented reversible phase transition through formation/cleavage of a weak but attractive B–H∙∙∙Cu(II) interactions. We provide evidence for the observed reversible process by a combination of single-crystal (SCXRD), powder (PXRD) X-ray diffraction, Raman spectroscopy and DFT calculations. This reversible transformation is achieved by solvent-guest exchange under ambient conditions in one direction and in DMF solutions at room temperature in the other. The transformation is mediated by B–H∙∙∙Cu(II) interactions, when generating open metal sites during solvent-exchange.

## 2. Results and Discussion

**Figure 1:**
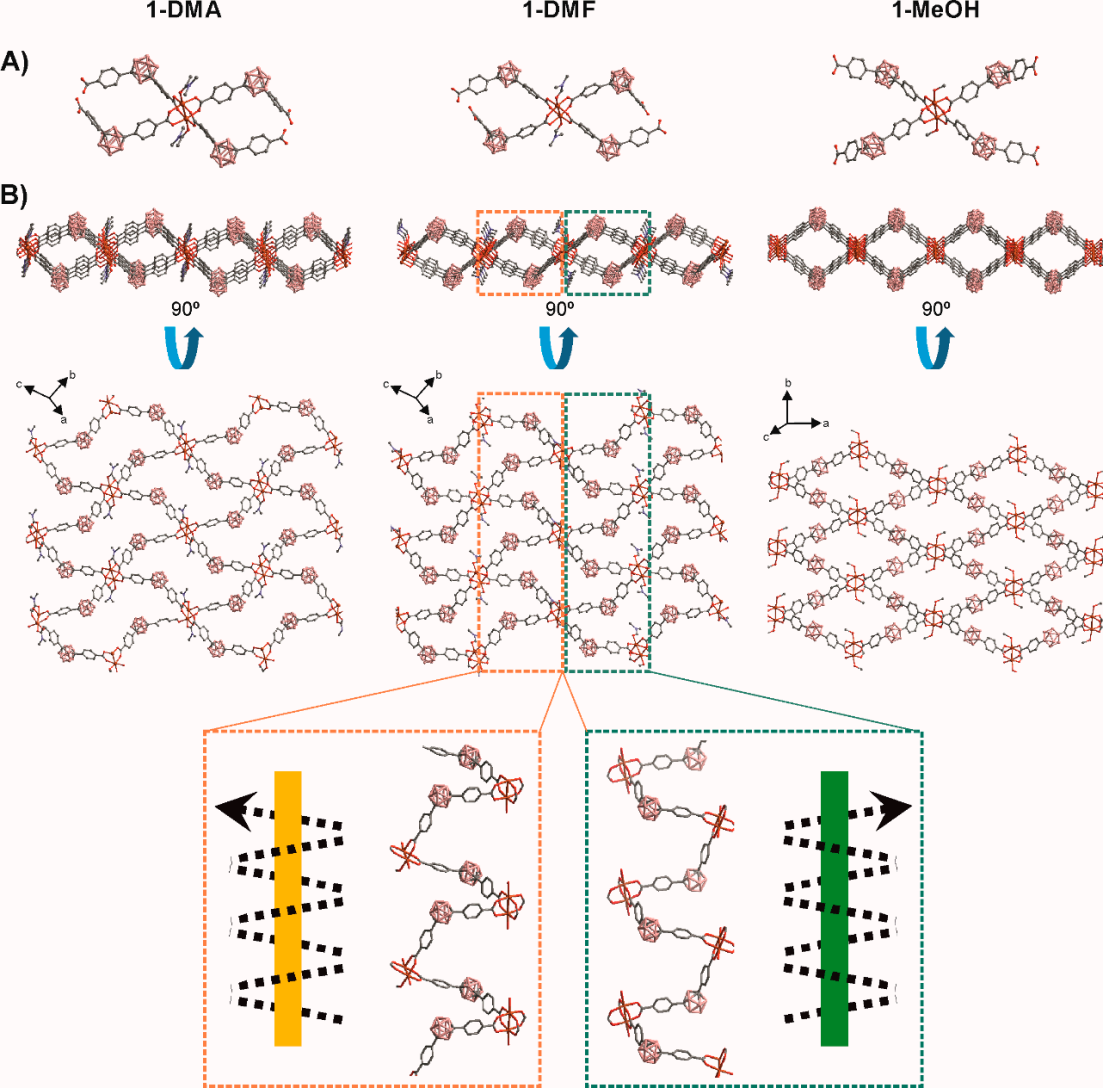
A comparative view of the crystal structures of 1-DMA, 1-DMF, and 1-MeOH. (**A**) View of the Cu2-paddlewheel units with L1 coordination. (**B**) Two perpendicular views of the extended structures showing the 2D 4^4^ networks; 1D helical chains with right hand (green) or left hand (orange) are indicated for 1-DMF. H atoms are omitted for clarity. Color code: B pink; C grey; O red; N blue, Cu orange.

Reaction of Cu(NO_3_)_2_, and L1 in dimethylformamide (DMF), dimethylacetamamide (DMA) or methanol (MeOH) at 80 °C for 48 h afforded greenish crystals for [Cu_2_(L1)_2_(Solv)_2_]•xSolv (1-Solv = 1-DMF, 1-DMA or 1-MeOH) in good yield. IR spectrum showed the characteristic broad B-H stretching bands from the carborane (in the range 2617–2531 cm^−1^), and the C=O vibration of the carboxylate groups (Figure S1). Single-crystal X-ray diffraction (Figure 1 and Tables S1 and S2) revealed a 2D network for all the compounds. Phase purity was confirmed by elemental analysis and powder X-ray diffraction (PXRD) for 1-DMF and 1-DMA (Figure S2). Crystals for 1-MeOH were only stable in the solvent and a rapid phase transition was observed when the crystals were dried in air (vide infra).

**Figure 2:**
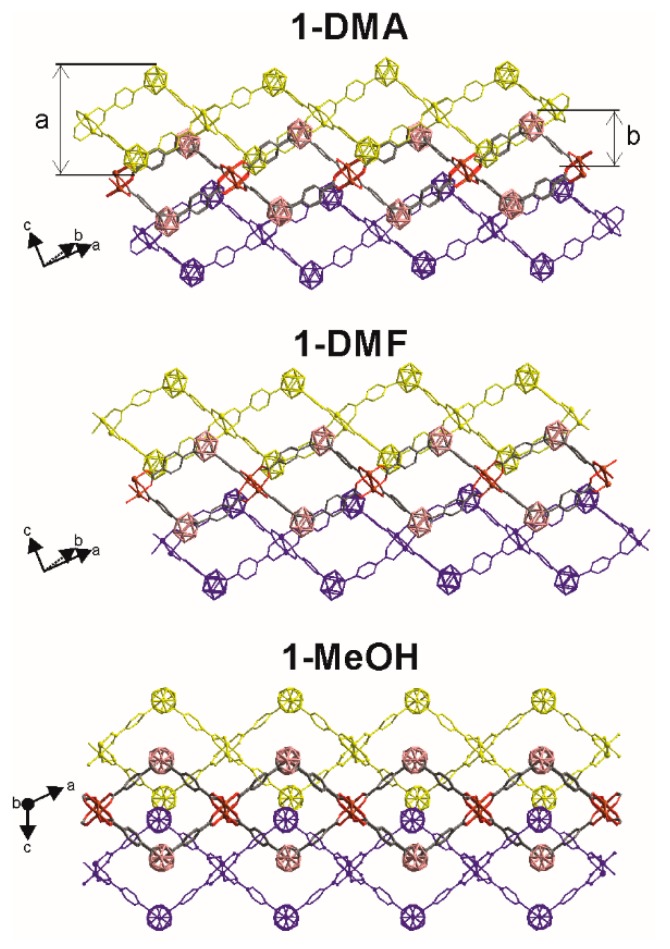
View of the packing structures for 1-Solv (Solv = DMA, DMF, and MeOH) showing the staking of 2D layer structures in which the various colors differentiates the contiguous layers. Coordinated solvent molecules are omitted for clarity. Layer thickness (a) and Interlayer distance (b) are only represented in 1-DMA. See Table 1 for values for all the structures.

The basic unit of 1-Solv is a Cu_2_-paddlewheel motif of [Cu_2_(COO)_4_] units (Figure 1). The Cu–Cu distances in the paddlewheel units are 2.647, 2.642 and 2.620 Å for 1-DMA, 1-DMF, and 1-MeOH, respectively. The two copper atoms share four L1 linkers at the basal positions and one oxygen atom from the solvent (DMA, DMF or MeOH for 1-DMA, 1-DMF, and 1-MeOH, respectively) occupy the apical positions (Figure 1A). Cu–OOC and Cu–O_solv_ bond lengths range from 1.952 to 1.971 Å and 2.081 to 2.137 Å, respectively. The carborane L1 linker shows a V-shape (OOC–CBcentroid–COO ≈ 111–115°) and two noncoplanar phenyl rings (78–87°). Thus, the bent L1 linkers and noncoplanar phenyl rings in 1-Solv adopt a 4^4^-grid topology by bending alternately above and below the plane containing the paddlewheel [Cu_2_(COO)_4_] units to produce a very corrugated 2D layers. As shown in Figure 1b, the 2D 4^4^ net consists of chair-like units, similar to that found in other related V-shape ditopic linkers [68,69,70]. Table 1 summarizes the parameters for each of the structures in this work and in comparison with some related structures where there is a 1,3-benzene moiety instead of a *m*-carborane unit [68,69,70]. In each 4^4^ unit in our structures, two consecutive carborane clusters are above the plane containing the paddlewheel [Cu_2_(COO)_4_] units, and the other two are below the plane. The corrugated layers in 1-Solv are thick (~15-16 Å, Table 1) and as can be seen in Figure 1B, they create nanoscale channels (see colored dotted rectangles in Figure 1B). A more detailed analysis of the structures shows that the nanoscale channels are formed by the particular bridging coordination of the V-shape L_CB_(COO)_2_ linker. Each carborane linker bridges two different paddlewheel units in a way that result in a single-strand helix chain (Figure 1), generating the above-mentioned channels. These 1D helical chains are alternately connected with each other through the paddlewheel units, constructing the observed 2D networks. The handedness of the 2_1_ helicity for all descriptions in this paper are determined using a method similar to the supramolecular tilt chirality method (*STC*) [71]. In brief, given a helix in front of the 2_1_ screw axis inclining to the right, the assemblies can be defined to be right-handed, or inclining to the left are defined to be left-handed. Quite interestingly, the pitches of the helixes are different in each of the new structures. The pitch decreases in the order 1-DMA (16.2) > 1-DMF (14.4) > 1-MeOH (12.5) and correlate with the shorter of the two diagonal paddlewheel to paddlewheel distances in each 4^4^ chair-like unit (Table 1). Such changes in the pitch suggest that a single 2D sheet would stretch or shrink, depending on the solvent. The analysis of data in Table 1 reveals that the larger diagonal paddlewheel to paddlewheel distances in each 4^4^ chair-like unit vary between 28–30 Å, whereas the variation for the shorter diagonal distances is much smaller (13–20 Å). Interestingly, the corresponding distances for the non-carborane containing CPs, e.g., those having a 1,3-benzene moiety instead of a *m*-carborane unit are longer (ca. 20 × 28 Å) [68,69,70]. A shorter *m*-carborane dicarboxylic ligand (1,7-dihydroxycarbonyl)-1,7-dicarba-*closo*-dodecaborane) provided related Cu-Paddlewheel-based 2D structures with smaller 4^4^ nets and shorter diagonal paddlewheel to paddlewheel distances (13–16 Å; Table 1) [44]. As mentioned above, the shorter of the two diagonal paddlewheel to paddlewheel distances in each 4^4^ chair-like unit corresponds to the pitch of the single-strand helix chains in the structures (Figure 1B). Larger pitches (or diagonal distances) for the non-carborane CPs favor interpenetration in all structures reported in the literature [68,69,70]. However, no interpenetration is observed for the structures reported in this work. As mentioned above, the pitch in 1-Solv increases with the size of the solvent molecules. The latter is followed by a concomitant decrease in the layer thickness, as it happens, for example, when one stretches or compresses a coil. Thus, the 1-DMA and 1-DMF layer thicknesses are 15.4 and 15.0 Å, respectively, whereas that for 1-MeOH is 16.3 Å. However, this does not seem to affect the interlayer distances as these are all very close (Table 1) and similar to other related 2D Cu(II) coordination polymers [72]. Stacking of the 2D layers gives rise to the 3D structures of the compounds. A comparison of the 3D structures shows only lateral displacements or sliding of the 2D layers as shown in Figure 2. Layers in 1-DMA and 1-DMF arrange in a way to maximize the nanoscale channels, whereas those in 1-MeOH are occluded.

**Figure 3:**
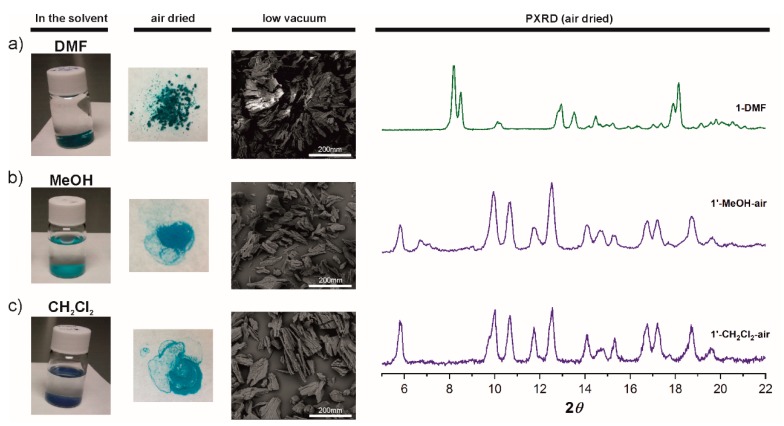
Left: Photographs and SEM images showing crystals color and morphology of as made 1-DMF (**a**), after 2 days in methanol (**b**) and after 2 days in dichloromethane (**c**), in the solvent and after being exposed to air. Right: PXRD patterns for 1-DMF, 1′-MeOH-air, and 1′-CH_2_Cl_2_-air.

Thermogravimetric analysis of 1-DMA and 1-DMF from room temperature to 600 °C shows a continuous drop until 400 °C (Figure S3), so that evaporation of the free and coordinated solvent cannot be distinguished from the decomposition of the framework, making the thermal activation difficult. Thus, solvent exchange was carried out successively with methanol (MeOH; 2 days) and dichloromethane (CH_2_Cl_2_ for 2 days; see Experimental for details) for 1-DMF. Treatment of large 1-DMF crystals with MeOH showed a clear macroscopic transformation in the size and color of the crystals to give a new phase 1′-MeOH (Figure 3 and videos in the supporting information). Large platelets broke into small pieces, followed with a color change from greenish to sky blue (Figure 3b). Immersion of 1′-MeOH crystals in CH_2_Cl_2_ provoked a color change from sky blue to deep navy (1′-CH_2_Cl_2_), consistent with the presence of OMS [24] but no further change of morphology of crystals was observed (Figure 3c). In contact with air, deep navy colored crystalline 1′-CH_2_Cl_2_ turns within seconds into sky blue color. TGA curves for air dried samples of 1′-MeOH-air and 1′-CH_2_Cl_2_-air show very similar profiles that are significantly different to that for 1-DMF; 1′-MeOH-air and 1′-CH_2_Cl_2_-air show a three-step loss, one at 75-98, 280 and another at 350 °C (Figure S4).

Remarkably, PXRD data for 1′-MeOH-air and 1′-CH_2_Cl_2_-air show that both correspond to the same phase and those are markedly different to that for 1-DMF (Figure 3). The PXRD data is consistent with a phase transition triggered by immersing 1-DMF in MeOH, the new crystalline phase remaining stable in CH_2_Cl_2_. Quite interestingly, the PXRD pattern for 1′-MeOH-air clearly differs from that for the calculated pattern of 1-MeOH (Figure S5). As mentioned above, crystals for 1-MeOH were only stable in the solvent and a rapid phase transition was observed when the crystals were dried in air; multiple single crystal measurement trials showed a fast change of symmetry on solvent loss. The new symmetry appears to be a centered version of the original cell with a doubling of the *a* parameter, diffraction was too poor to yield more than a rough backbone structure with very high R values. PXRD of the latter crystals exposed to air confirmed that 1-MeOH transformed into 1′-MeOH-air (Figure S5). Unfortunately, we could not obtain the SCXRD structure of either 1′-MeOH-air or 1′-CH_2_Cl_2_-air even by using synchrotron radiation measurements. Nevertheless, elemental analyses correlate with 1′-MeOH-air and 1′-CH_2_Cl_2_-air being [Cu_2_(L1)_2_(H_2_O)(2MeOH)] [73] and [Cu_2_(L1)_2_(H_2_O)(CH_2_Cl_2_)], respectively. Overall the data suggest the successive exchange of Cu-bound DMF by MeOH and then CH_2_Cl_2_ to provide the deep navy colored 1′-CH_2_Cl_2_-wet having OMS (see Raman discussion bellow), that on exposure to air rapidly changes to sky blue. As mention in the introduction, such chemical route to active OMS species have been widely investigated and showed that owing to the weak Lewis basicity of CH_2_Cl_2_, it spontaneously dissociated from the metal and thus provided open metal sites at room temperature [21,23,25,26]. However, we note that whereas the color change from deep navy to sky blue on moist water coordination usually occurs within minutes or hours in the reported examples [25,74], color change from 1′-CH_2_Cl_2_-wet to 1′-CH_2_Cl_2_-air takes place within seconds (*vide infra*). Based on the observed color changes and Raman data bellow, we hypothesize 1′-CH_2_Cl_2_-wet being [Cu_2_(L1)_2_]xCH_2_Cl_2_, where the CH_2_Cl_2_ solvent molecules are partially or totally uncoordinated.

**Figure 4:**
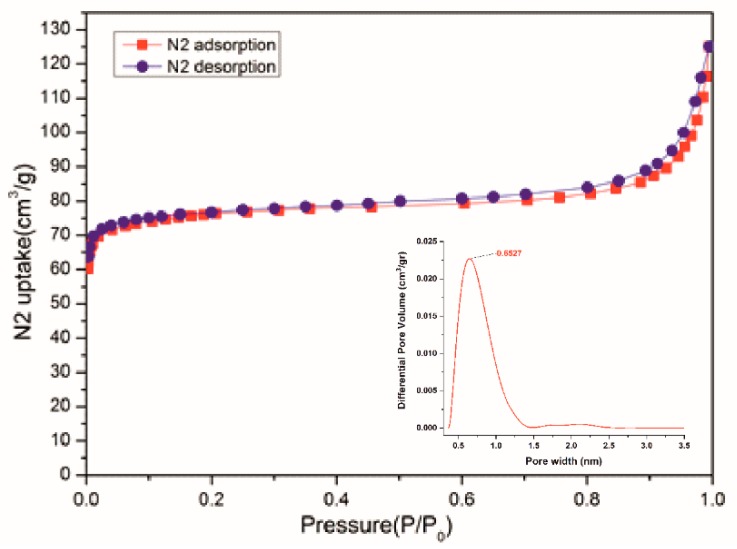
N_2_ adsorption isotherm at 77 K for activated 1′-CH_2_Cl_2_-air. The inset shows the calculated pore-size distribution using a 2D-NLDFT Heterogeneous Surface model.

1′-CH_2_Cl_2_-air was activated at 120 °C under dynamic high vacuum for 20 h, affording a deep navy solid, presumably corresponding to desolvated [Cu_2_(L1)_2_] (1′-activated), containing OMS. The latter is porous to N_2_ at 77 K (BET surface area: 301 m^2^) and also to CO_2_ (41 cm^3^ g^−1^ STP) at 273 K and 1 bar. N_2_ sorption-desorption isotherms exhibited a typical reversible type-I behavior with an uptake of 125 cm^3^/g (Figure 4). The pore width distribution was 6–7 Å (Figure 4 and Figure S8), suggesting that the micropores from 1′-activated might correlate with the observed nanoscale channels in 1-solv (Figure 1). Interestingly, CO_2_ sorption capacity of 1′-activated is twice that measured for the related (but shorter) carborane-dicarboxylated Cu_2_-paddle wheel CP [44].

**Figure 5:**
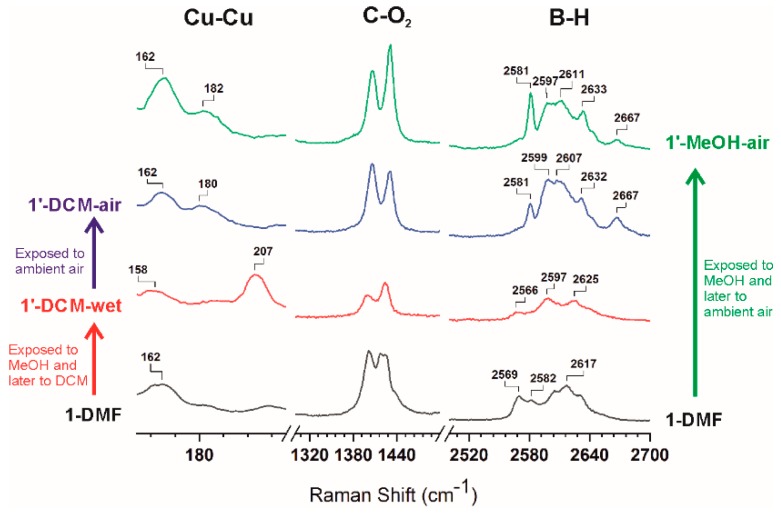
Successive changes in the Raman spectrum of 1-DMF after sequential exposure to methanol (MeOH), dichloromethane (CH_2_Cl_2_) and ambient air (See full spectra in the SI).

Having determined that [Cu_2_(L1)_2_] (1′-activated) retains porosity, we evaluated the possible structural reversibility. Indeed, 1-DMF could be obtained by immersing samples of 1′-activated, 1′-CH_2_Cl_2_-air or 1′-MeOH-air into DMF at room temperature (Figure S6). Such a reversible transformation under ambient conditions indicates a rather weak interaction between layers in 1′ structures. Intrigued by this reversible phase transition, we further investigated the process by Raman spectroscopy in order to identify the possible changes in the paddlewheel units in 1. Therefore, we measured the Raman spectra for 1-DMF, 1′-MeOH-air, 1′-CH_2_Cl_2_-air, and 1′-CH_2_Cl_2_-wet. A comparison of the complete and selected Raman spectra for all the above compounds is provided in Figure S9 and Figure 5, respectively. Stretching modes of Cu–Cu vibrations (240–150 cm^−1^) were clearly observed in all the samples as broad bands. Whereas Cu–Cu vibration for 1-DMF appeared at 162 cm^−1^, that for 1′-CH_2_Cl_2_-wet appeared at 207 cm^−1^, that is, blue-shifted ca. 50 cm^−1^. Such blue-shift displacement correlates well with that observed for HKUST-1 on chemical activation [26]. As mentioned earlier, exposure of 1′-CH_2_Cl_2_-wet to air quickly provided 1′-CH_2_Cl_2_-air. Interestingly, after the sample was exposed to ambient air (within 1 minute) a color change from navy to sky blue was noticed and the Cu–Cu vibration observed for 1′-CH_2_Cl_2_-wet at 207 cm^−1^, was red-shifted and split into two bands at approximately 180 and 162 cm^−1^ in 1′-CH_2_Cl_2_-air (Figure 5). The latter remained unaltered with longer exposure to air. Two bands are also observed in the Raman spectrum of 1′-MeOH-air, at 182 and 162 cm^−1^. The observed spontaneous red shift on exposing 1′-CH_2_Cl_2_-wet to air is consistent with a lengthening of the Cu∙∙∙Cu distances on dissociation of weakly coordinated CH_2_Cl_2_ and coordination of other better bonding species, such as e.g., H_2_O from air [23]. The above data is in agreement with previously reported data and proof that DMF-coordinated to Cu in 1 can be effectively and successively dissociated by MeOH and CH_2_Cl_2_ [24]. It is noteworthy that no other changes in the Raman signals were observed in the region for the organic moiety of the linker (Figure 5 and Figure S9). The characteristic double band of *ν*_sym_(COO^−^) vibration for the bridged carboxylates at around 1400 and 1420 cm^−1^ (Figure 5 and Figure S9) remains mostly unchanged, demonstrating that there is no significant change in the coordination mode of the carboxylates from the Cu_2_-paddlewheel units during the transformation. There is, however, a clear change in the B-H vibrations, suggesting that the change in the Cu∙∙∙Cu distances also affects the carborane moieties. Thus, B-H vibrations appear as broad bands at 2569, 2582 and 2617 cm^−1^ in 1-DMF and change into three broad bands at 2566, 2597 and 2625 cm^−1^ in 1′-CH_2_Cl_2_-wet (Figure 5). A clear change was observed when 1′-CH_2_Cl_2_-wet was exposed to the air, providing 1′-CH_2_Cl_2_-air. Thus, new B-H vibrations appear at 2581, 2599, 2607, 2632 and 2667 cm^−1^ for 1′-CH_2_Cl_2_-air. As observed in the Cu–Cu vibration region, a nearly identical Raman spectrum to that of 1′-CH_2_Cl_2_-air is observed in the case of 1′-MeOH-air.

A deep understanding of the vibrational features described in the present work has been obtained by density functional theory (DFT) calculations (See the experimental section for details). In order to simplify the calculations, we used model structures consisting of four monosubstituted carborane-based ligands bonded to a Cu_2_-paddlewheel unit [Cu_2_(L_CB_)_4_(Solv)_2_] (L_CB_ = 1-(4-carboxyphenyl)-1,7-dicarba-*closo*-dodecaborane; Solv = DMF, MeOH, H_2_O, C_2_B_10_H_12_ and none; see Table 2, model compounds). We computed bond distances, vibrational frequencies and energies for paddlewheel units having DMF, MeOH, H_2_O, none and/or a discrete *m*-carborane moiety bonded to the Cu atoms at the apical positions (Table 2). Calculation for the Cu–Cu vibrational frequencies for 1-DMF resulted in a frequency of 207 cm^−1^, a value significantly higher than the experimental value (162 cm^−1^). This disagreement in the Cu–Cu calculated values was also observed in models for HKUST-1 [25]. The calculations, however, correlate well with the observed raman shifts (*vide infra*), that is, the vibrational frequencies increases as the coordination strength of Lewis base molecules decreases and viceversa. The calculated Raman spectra show a good agreement with the experimental data (Figure S10). Regardless of the expected shift between the experimental and calculated Raman spectra, the model provides reliable results in the range 300–3200 cm^−1^ and allowed identification of all the experimental bands (Figure S10). Quite remarkably, the relatively sharp band at 2581 cm^−1^ in 1′-CH_2_Cl_2_-air and 1′-MeOH-air (Figure 5) can be attributed to a B–H∙∙∙Cu(II) agostic interaction, by comparison with our models where at least one hydride of a *m*-carborane molecule is coordinating to one of the Cu(II) atoms of the paddlewheel unit. In addition, the observed weak band at 2667 cm^−1^ seems to correlate with one of the B–H hydrides in close proximity (although not bonded) to the Cu(II) atoms in the model. Comparison of the B–H Raman region for 1′-CH_2_Cl_2_-air and 1′-MeOH-air (Figure 5) with that for various models (Figure S11) indicates that interactions between 2D layers involve one or perhaps two B–H∙∙∙Cu(II) interaction per paddlewheel unit. It is possible that not all Cu(II) atoms in each paddlewheel unit are interacting with a hydride, leaving this open to coordinate one water molecule from the air or even remain unsaturated (OMS). In fact, the corrugated shape of the 2D layers might complicate the match between all OMSs and available hydrides between layers. Further evidence for such B–H∙∙∙Cu(II) interactions is obtained from the DFT calculations on our model compounds. An energy comparison of our DFT models (Table 2) reveals a clear stabilization of the fully open metal site model [Cu_2_(L_CB_)_4_] when interacting with one or two B–H hydrides. Thus, stabilization energies of 34.4, 26.7 or 13.6 Kcal/mol were obtained for [Cu_2_(L_CB_)_4_] interacting with one H_2_O and one *m*-carborane molecule, two *m*-carborane molecules or one OMS and one *m*-carborane molecule, respectively. In addition, calculated Cu–Cu vibrational frequencies for our models (Table 2) show the same trend that was observed experimentally (Figure 5), that is, a blue-shift (from lower to higher frequencies) on generation of open metal sites (or removal of DMF by CH_2_Cl_2_ from 1-DMF) and red-shift displacements (from higher to lower frequencies) on solvent or B-H coordination. Assuming that the observed trend is correct, we hypothesize that the experimentally observed Cu–Cu bands at 162 and 180 cm-1 for 1′-CH_2_Cl_2_-air and 1′-MeOH-air correspond to B–H∙∙∙Cu–Cu∙∙∙H–B and B–H∙∙∙Cu–Cu(OMS) species, although we cannot exclude the formation of partially hydrated species. In fact, it seems difficult that all Cu atoms are coordinated to a hydride atom, due to the high corrugation of 2D layers. It is also possible that multiple (B–H)_x_∙∙∙Cu interactions are taking place. This will certainly stabilize the binding enthalpy of such interactions [75]. A reversible coordination of B–H to a Cu(I) complex has been previously reported for a molecular system [75]. 2D CPs or MOFs present the unique feature of structural isomerism by sliding of layers involving the supramolecular interactions between them and the included solvent [76,77]. However, to our knowledge, no such mediated B–H∙∙∙Metal transformations have been observed previously in coordination polymers or MOFs. The observed lower stability of crystals for 1-MeOH than the corresponding ones for 1-DMF (Figure 1), when exposed to air, can be explained by a comparable binding energy for B–H∙∙∙Cu and Me(H)O∙∙∙Cu interactions.

## 3. Materials and Methods

### 3.1. Characterization and Methods

Attenuated total reflection Fourier transformed infra-red (ATR-FTIR) spectra were recorded using a PerkinElmer Spectrum One spectrometer equipped with a Universal ATR sampling accessory. Spectra were collected with 2 cm^−1^ spectral resolution in the 4000–650 cm^−1^ range. Elemental analyses were obtained by using a Thermo (Carlo Erba) Flash 2000 Elemental Analyser, configured for wt% CHN. Thermogravimetric Analysis (TGA) was performed in N_2_, on an nSTA 449 F1 Jupiter-Simultaneous TGA-DSC or SDT Q600 V8.3 Build 101 instruments (heating rate: 10 °C/min; temperature range: 25 °C to 600 °C). Gas sorption-desorption (CO_2_/273 K and N_2_/77 K) measurements were performed using an ASAP2020 surface area analyzer. Samples were first degassed at 120 °C for 20 h. Powder X-ray Diffraction (PXRD) was recorded at room temperature on a Siemens D-5000 diffractometer with Cu Kα radiation Field-emission(λ = 1.54056 Å, 45kV, 35mA, increment = 0.02^o^). Morphological features were examined first by optical microscopy and next by scanning electron microscopy (SEM) with a QUANTA FEI 200 FEGESEM microscope. *Raman spectra were recorded* using an LabRam HR800 (Horiba Jobin-Yvon) dispersive mircro-raman spectrometer coupled with a CCD *detector.* A solid state laser with a 532 nm wavelength was used as the excitation source and the spectra were measured in a backscattering configuration through an Olympus BXFM objective. Excitation of the samples was performed by focusing the laser beam on crystalline samples with a laser power of 0.5 mW with a 50x magnifying objective lens.

### 3.2. Materials

1,7-di(4-carboxyphenyl)-1,7-dicarba-*closo*-dodecaborane ligand was synthesized according to the literature procedure [78]. CPs syntheses were done in air. All chemicals were commercially available and used as received.

Synthesis of [Cu_2_(L1)_2_(DMF)_2_]·2DMF·H_2_O (1-DMF). Cu(NO_3_)_2_·6H_2_O (48.7 mg, 0.167 mmol) was mixed with H_2_L1 (64.1 mg, 0.167 mmol) in 4 mL of DMF. This mixture was sonicated until all solids were uniformly dissolved, followed by heating at 80 °C for 48 h. Greenish crystals of 1-DMF were collected and washed with DMF (60 mg, 59.8%). IR (ATR; selected bands; cm^−1^): 2605, 2570 (BH); 1672 (C=O from DMF); 1617 (C=O from carboxylate). Elemental analysis (%) calculated for Cu_2_(L_CB_(COO)_2_)_2_(DMF)_4_(H_2_O): C 43.96, H 5.53, N 4.66; Found: C 43.91, H 5.58, N 5.20.

Synthesis of [Cu_2_(L1)_2_(DMA)_2_]·DMA·H_2_O (1-DMA). Cu(NO_3_)_2_·6H_2_O (48.7 mg, 0.167 mmol) was mixed with H_2_L1 (64.1 mg, 0.167 mmol) in 4 mL of DMA. This mixture was sonicated until all solids were uniformly dissolved, followed by heating at 80 °C for 48 h. Greenish crystals of 1-DMA were collected and washed with DMA (55.0 mg, 56.2%). IR (ATR; selected bands; cm^−1^): 2604 (BH); 1646 (C=O from DMA); 1602 (C=O from carboxylate). Elemental analysis (%) calculated for Cu_2_(L_CB_(COO)_2_)_2_(DMA)_3_(H_2_O): C 45.12, H 5.59, N 3.59; Found: C 44.52, H 5.51, N 4.02.

[Cu_2_(L1)_2_(MeOH)_2_]·4MeOH (1-MeOH). Cu(NO_3_)_2_·6H_2_O (48.7 mg, 0.167 mmol) was mixed with H_2_L1 (64.1 mg, 0.167 mmol) in 4 mL of MeOH. This mixture was sonicated until all solids were uniformly dissolved, followed by heating at 80 °C for 24 h. Light-blue crystals of 1-MeOH were collected, washed with MeOH and stored in this solvent (46 mg, 50.8%).

General method for chemical activation of 1-DMF. As-synthesized greenish crystals of 1-DMF (10 mg) were immersed in methanol (10 mL), provoking 1-DMF brake into smaller sky blue crystals of 1′-MeOH. MeOH was further exchanged once a day for 2 days and replaced by CH_2_Cl_2_ (10 mL). The latter provided deep blue crystals for 1′-CH_2_Cl_2_-wet after 10 min. The entire process was performed while maintaining the crystals fully covered with the solvents. When deep blue crystals for 1′-CH_2_Cl_2_-wet were dried on filter paper at room temperature a very fast color change was observed to sky blue crystals for 1′-CH_2_Cl_2_-air. See videos for transformation and color changes.

1′-MeOH-air: IR (ATR; selected bands; cm^−1^): 2596, 2579 (BH); 1610 (C=O from carboxylate). Elemental analysis (%) calculated for Cu_2_(L_CB_(COO)_2_)_2_(H_2_O)(2MeOH): C 41.93, H 4.76; Found: C 41.99, H 4.70.

1′-CH_2_Cl_2_-air: IR (ATR; selected bands; cm^−1^): 2596, 25 (BH); 1610 (C=O from carboxylate). Elemental analysis (%) calculated for Cu_2_(L1)_2_(H_2_O)(CH_2_Cl_2_): C 39.84, H 4.05; Found: C 39.71, H 4.26.

Synthesis of 1-DMF from 1′-Solv-air. Crystals for freshly made 1′-Solv-air (10 mg; Solv = MeOH or CH_2_Cl_2_) were suspended in DMF (10 mL) in a capped glass vial and left at room temperature for 3 days. No dissolution of the crystals was observed during this time. The obtained crystals for 1-DMF were then filtered, washed with DMF and stored in the same solvent. See Figure S10 for powder X-ray diffraction.

### 3.3. Crystallography

Measured crystals were prepared under inert conditions immersed in perfluoropolyether or paratone as protecting oil for manipulation. Suitable crystals were mounted on MiTeGen Micromounts^TM^, and used for data collection. Crystallographic data for 1-DMA were collected with a Bruker D8 Venture diffractometer, processed with APEX3 program [79] and corrected for absorption using TWINABS [80]. The structures were solved by direct methods and subsequently refined by correction of F^2^ against all reflections. All non-hydrogen atoms were refined with anisotropic thermal parameters by full-matrix least-squares calculations on F^2^ [81]. Hydrogen atoms were not located in difference Fourier maps and included as fixed contributions riding on attached atoms with isotropic thermal displacement parameter 1.2 (C–H, B–H) or 1.5 (O–H) times those of the respective atom. 1-DMA was treated as a two component non-merohedral twin. The twin matrix describes a rotation of 179.8° around the [1-1 0] direction, given by the matrix (0-1 0 -1 0 0 0 0-1). The structure of 1-DMA was solved using direct methods with only the non-overlapping reflections of component 1. The structure was refined using the HKLF 5 routine with all reflections of component 1 (including the overlapping ones), resulting in a BASF value of 0.2356(12). The structure exhibits disorder of the coordinated DMA molecule, which was successfully refined using a two-site model with 0.63:0.37 occupancy ratio. The contribution of the disordered solvent molecules to the diffraction pattern could not be rigorously included in the model and were consequently removed with the SQUEEZE routine of PLATON [82] that suggest the presence of 2 DMA molecules that have not been included in the refined structure but considered for calculation of empirical formula, formula weight, density, linear absorption coefficient and F(000).

Crystallographic data for 1-DMF and 1-MeOH were collected on a Rigaku AFC12 4-circle goniometer equipped with a HyPix 6000HE (Hybrid Photon Counting) detector mounted at the window of an FR−E+ SuperBright molybdenum (Mo Kα1/Kα2 = 0.71073Å) rotating anode generator with VHF Varimax optics (70 µm focus) operating at 2.475 kW (45 kV, 55 mA). Data reduction was performed using the CrysAlisPro (Rigaku, V1.171.40.18b, 2018) software and the structure was solved by Intrinsic Phasing using the ShelXT (Sheldrick, 2015) structure solution program and refined by Least Squares using version 2016/6 of ShelXL (Sheldrick, 2015). In the case of 1-MeOH the crystal was a non-merohedral twin and data integrated from one component were used for the refinement. Discarding severely overlapped reflections has led to a low completeness. A summary of crystal data is reported in Table S1.

### 3.4. Computational Details

The geometry of all computed systems was optimized by dispersion-corrected (D3) [83] density functional theory (DFT) using the hybrid B3LYP functional [84,85,86] as implemented in the Gaussian 09 [87]. The Cu atom was described using the scalar relativistic Stuttgart-Dresden SDD pseudopotential and its associated double-ζ basis set, [88] complemented with a set of *f* polarization functions [89]. The 6–31G(d) basis sets was used for the rest of atoms [90]. All stationary points were characterized and confirmed by frequency analysis.

Calculations were performed on molecular structures according to the general formula [Cu_2_(L_CB_(COO)_2_)_2_(Solv)_2_] where L_CB_(COO)_2_ is 1,7-di(4-carboxyphenyl)-1,7-dicarba-*closo*-dodecaborane. Several Solv were considered occupying two coordination sites to investigate their effect on the properties of the system. The Solv molecules computed were DMF, H_2_O, MeOH and *closo*-dodecaborane.

The electronic state of the system was initially investigated for Solv = DMF and MeOH. The closed shell singlets were calculated to be 35.8 kcal/mol and 35.4 kcal/mol higher in energy than the triplets for the complexes with DMF and MeOH as Solv, respectively. Nevertheless, the open shell singlets states were calculated to be lower in energy than the triplet state by 0.6 kcal/mol for both complexes. This is in agreement with previous DFT studies in the literature for copper paddlewheel-based systems [91,92,93,94,95]. Thus, all the structures presented in this work were calculated as open shell singlets.

## 4. Conclusions

In summary, we report the syntheses of 2D 1-Solv (Solv: DMF, MeOH or CH_2_Cl_2_), a novel family of Cu_2_-paddlewheel CPs that incorporate the flexible ligand 1,7-di(4-carboxyphenyl)-1,7-dicarba-*closo*-dodecaborane (L1). 1-DMF undergoes a reversible phase transition on solvent exchange and provided a crystalline material that is porous to N_2_ and CO_2_. The combination of the experimental and calculated data supports the spontaneous release of the solvent from 1-DMF, with the consequent generation of OMSs. Sliding of the layers allows the OMSs to be in close proximity to readily available hydride atoms of the many present from the carborane moieties, and thus promote the formation of B–H∙∙∙Cu(II) interactions. The observed phase transition on solvent loss is accompanied by new Raman modes in the B–H and Cu–Cu region that are in agreement with the formation of B–H∙∙∙Cu(II) interactions. There is enough flexibility in the solid to move the paddlewheels relative to each other so that the Cu centers can interact with the many available hydride atoms from the carborane moieties in another paddlewheel unit. Such B–H∙∙∙Cu(II) interactions can be easily disturbed in the presence of a strongly coordinating solvent such as DMF and provide the starting 1-DMF structure at room temperature. The present work adds unprecedented knowledge to the possible reasons for boranes or carboranes acting to stabilize flexible MOFs [48] but it also discloses another possible mechanism for constructing new flexible architectures or hydride/MOF composites.

## Figures and Tables

**Table 1:** Selected crystal parameters for compounds in this work and related 2D structures.

Coordination Polymer	SG*^a^*	D*^b^*	4^4^ nets Diagonal Distances (Å)*^c^*	*P* (Å)*^d^*	Layer Thickness*^e^* (Å)	Interlayer Distances (Å)
1-DMA (this work)	*P*-1	1.262	16.2 × 29.0	16.2	15.41	6.94
1-DMF (this work)	*P*-1	1.355	14.4 × 29.4	14.4	14.95	6.86
1-MeOH (this work)	*P*2_1_/*c*	1.367	12.5 × 29.8	12.5	16.29	7.06
PELFOE*^f^* ([CuL^1^∙DMA]DMF)	*C*2/*c*	1.448	19.7 × 27.6	19.7	-	interpenetrated
MAPLAT*^f^* ([CuL^1^∙DMA]H2O)	*C*2/*c*	1.353	19.9 × 27.6	19.9	-	interpenetrated
OFEHOY*^f^* ([CuL^2^∙DMF]DMF)	*C*2/*c*	1.245	19.6 × 27.7	19.6	-	interpenetrated
NEYRIU*^f^* ([CuL^3^∙MeOH]MeOH)	*C*2/*c*	1.261	12.8 × 13.8	12.8	10.313	10.187
NEYREQ*^f^* ([CuL^3^∙MeOH]DMF)	*P*2_1_/*n*		12.7 × 14.0	12.7	9.827	10.454

*^a^* Space groups. *^b^* Calculated density. *^c^* Paddlewheel to paddlewheel distances in each in each 4^4^ chair-like unit. *^d^* Pitches of the 2_1_ helices. *^e^* Measured from H atom to H atom in B-H bonds; See also Figure 2. *^f^* CSD Refcodes. L^1^: 1,3-di(4-carboxyphenyl)benzene; L^2^: 5′-methyl-1,1′:3′,1”-terephenyl-4,4”-dicarboxylic acid; L^3^: 1,7-dicarba-closo-dodecaborane-1,7-dicarboxylato.

**Table 2:** Calculated Energies, Cu-Cu Bond Lengths and vibrational frequencies for the Model compounds [Cu_2_(L_CB_)_4_(Solv)_2_] (LCB = 1-(4-carboxyphenyl)-1,7-dicarba-closo-dodecaborane; Solv = DMF, MeOH, H_2_O, C_2_B_10_H_12_ and none). See experimental section for details.

Model Compounds (Molecules at the Apical Positions)	Energy^a^ (Kcal/mol)	Cu-Cu Distances (Å)		Vibrational Cu-Cu Frequencies (cm^−1^)	
		Exp.	Calc.	Exp.	Calc.
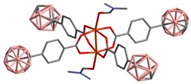 (2 Molecules of DMF)	−41.6	2.641^b^	2.668	162^b^	207
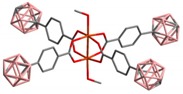 (2 Molecules of MeOH)	−41.0	2.620^c^	2.597	-	194
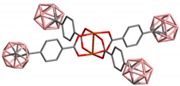 (None)	0	-	2.507	-	223
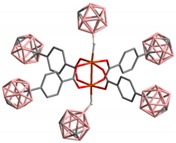 (2 Molecules of *m*-Carborane)	−26.7	-	2.561	-	206
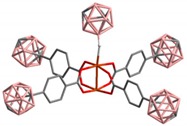 (1 Molecule of *m*-Carborane)	−13.6	-	2.536	-	214
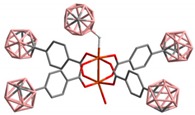 (1 Molecule of *m*-Carborane 1 molecule of H_2_O)	−34.4	-	2.577	-	192

^a^ See DFT calculation section for details. ^b^ For 1-DMF. ^c^ For 1-MeOH.

## Supplementary Materials

The following are available online. Table S1: Crystal and structure refinement data for 1-Solv (Solv = DMA, DMF or MeOH), Table S2: Selected distances (Å) and angles (°) for 1-Solv (Solv = DMA, DMF or MeOH), Figure S1. FT-IR spectra of L1 ligand, 1-DMA, 1-DMF, 1′-MeOH-air and 1′- CH_2_Cl_2_-air, Figure S2: Comparison of calculated and experimental PXRD fot 1-DMF and 1-DMA, Figure S3: Figure S3. TGA diagram of as made 1-DMA (red) and 1-DMF (green), Figure S4: TGA diagrams of 1′-MeOH-air (black) and 1′-CH_2_Cl_2_-air (red), Figure S5: PXRD comparison of calculated 1-MeOH, 1-MeOH-air and 1′-MeOH-air, Figure S6: PXRD patterns showing reversible transformation from different samples to 1-DMF, Figure S7: CO_2_ adsorption isotherm at 273 K for activated 1′- CH_2_Cl_2_-air, Figure S8: Pore Distribution by Density Functional Theory Model: CO_2_-DFT Slit Geometry for activated 1′- CH_2_Cl_2_-air, Figure S9: Comparison of Experimental Raman Spectra of 1-DMF, 1′-DCM-wet, 1′-DCM-air and 1′-MeOH-air, Figure S10: Comparison of Experimental and Calculated Raman for 1-DMF with [Cu_2_(LCB)_4_(DMF)_2_] (LCB = 1-(4-carboxyphenyl)-1,7-dicarba-closo-dodecaborane), Figure S11: Comparison of Experimental and Calculated Raman for 1′-CH_2_Cl_2_-air and 1′-MeOH-air with [Cu_2_(LCB)_4_(Solv)_2_] (LCB = 1-(4-carboxyphenyl)-1,7-dicarba-closo-dodecaborane; Solv = H_2_O, C_2_B_10_H_12_ and/or none (Open Metal Site)), computational details, Video S1: DMF plus MeOH, Video S2: DCM-wet plus DMF, Video S3: DCM-wet plus air.
