# Supplementary material for: A Reversible Phase Transition of 2D Coordination Layers by B–H∙∙∙Cu(II) Interactions in a Coordination Polymer
Source: Molecules. 2019 Sep 3;24(17):3204. doi: 10.3390/molecules24173204 (PMC6751501; doi:10.3390/molecules24173204)
Supplement: Supplementary file 1 [file molecules-24-03204-s001.zip › molecules-565909-supplementary/molecules-565909 - Supplementary final/molecules-565909-supplementary final.pdf]

Supplementary Information

A Reversible Phase Transition of 2D Coordination Layers by B–H···Cu(II) Interactions in a Coordination Polymer

Lei Gan [1], Pol G. Fonquernie [1], Mark E. Light [2], Gantulga Norjmaa [3], Gregori Ujaque [3], Duane Choquesillo-Lazarte [4], Julio Fraile [1], Francesc Teixidor [1], Clara Viñas [1], and José G. Planas [1,*]

[1] Institut de Ciència de Materials de Barcelona (ICMAB-CSIC), Bellaterra 08193, Spain
[2] Department of Chemistry, University of Southampton, Highfield, Southampton SO17 1BJ, UK.
[3] Departament de Química, Universitat Autònoma de Barcelona, 08193 Cerdanyola del Valles, Catalonia, Spain
[4] Laboratorio de Estudios Cristalográficos, IACT, CSIC-Universidad de Granada, Av. de las Palmeras 4, E-18100 Armilla, Granada, Spain
* Correspondence: jginerplanas@icmab.es

Academic editors: Igor B. Sivaev, Narayan S. Hosmane and Bohumír Grűner
Received: 19 July 2019; Accepted: 01 September 2019; Published: date

Table S1. Crystal and Structure Refinement data for 1-Solv (Solv = DMA, DMF or MeOH).			
Compound	**1-DMA**	**1-DMF**	**1-MeOH**
Empirical formula	$C_{48}H_{72}B_{20}N_4O_{12}Cu_2$	$C_{44}H_{64}B_{20}N_4O_{12}Cu_2$	$C_{38}H_{60}B_{20}O_{14}Cu_2$
Formula weight	1240.37	1184.27	1084.14
Crystal system	Triclinic	Triclinic	Monoclinic
Space group	P-1	P-1	P21/c
CCDC ref	1901504	1899491	1859468
Wavelength (Å)	Cu Kα (1.5418)	Mo Kα (0.71073)	Mo Kα (0.71073)
a (Å)	15.2205(5)	14.4079(13)	16.2198(5)
b (Å)	15.5001(6)	14.5172(12)	12.5200(3)
c (Å)	15.5639(5)	15.4994(11)	14.1322(5)
α (deg)	82.435(2)	86.242(6)	90
β (deg)	87.787(2)	89.578(7)	113.426(4)
γ(deg)	63.755(2)	63.842(8)	90
V ($Å^3$)	5897(2)	2901.8(4)	2633.30(16)
Z	2	2	2
F (000)	1284	1220.0	1116.0
θ (range)	2.865 - 67.266	3.735 - 25.027	2.126-28.499
Max./min. transmission	0.752817/ 0.615490	1.00000/0.59033	1.000/0.798
Ind refln (R_{int})	11538 (-)	10188 (-)	5966 (0.0714)
R1 (I > 2(I))<br>R1 (all data)<br>wR2<br>Wr2 (all data)	0.0469<br>0.0604<br>0.1122<br>0.1191	0.0863<br>0.1603<br>0.1718<br>0.2062	0.0455<br>0.0576<br>0.1220<br>0.1268

**Table S2**. Selected distances (Å) and angles (º) for **1-Solv** (**Solv** = **DMA**, **DMF** or **MeOH**).					
Compound	**Cu–Cu**	**Cu–O$_2$C**	**Cu–Osolv**	**O$_2$C–CB$_{centroid}$–CO$_2$**[a]	**Ph–Ph**[b]
**1-DMA**	2.643<br>2.647	1.952<br>1.959<br>1.961<br>1.962<br>1.964<br>1.965<br>1.977<br>1.983	2.081<br>2.124	112.62<br>115.43	86.76<br>86.45
**1-DMF**	2.607(2)<br>2.641(2)	1.941(4)<br>1.948(4)<br>1.959(5)<br>1.966(4)<br>1.972(4)<br>1.974(4)<br>1.981(5)<br>1.991(4)	2.111(5)<br>2.138(4)	110.54<br>114.98	82.21<br>77.7(2)
**1-MeOH**	2.620(50)	1.951(2)<br>1.958(2)<br>1.962(2)<br>1.983(2)	2.130(2)	112.22	87.29(30)

[a] Carboxylated Carbon to carborane centroid angle. [b] Angle between C_6H_4 rings in the $L_{CB}(COO)_2$ ligand.

Figure S1. **FT-IR spectra of L1 ligand, 1-DMA, 1-DMF, 1'-MeOH-air and 1'-CH_2Cl_2-air.**

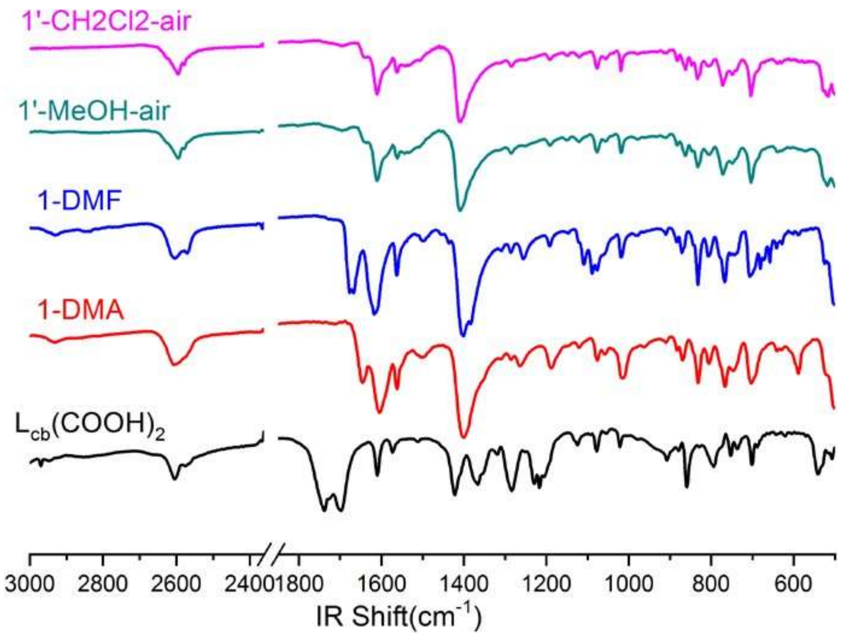

Figure S2. **Comparison of calculated and experimental PXRD fot 1-DMF and 1-DMA.**

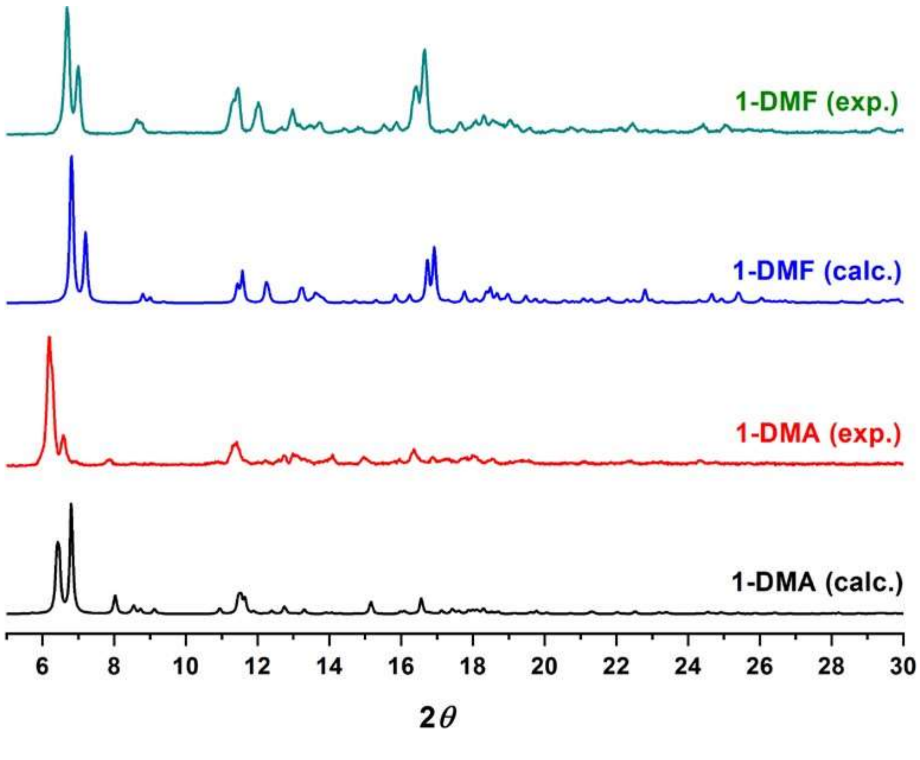

Figure S3. **TGA diagram of as made 1-DMA (red) and 1-DMF (green)**.

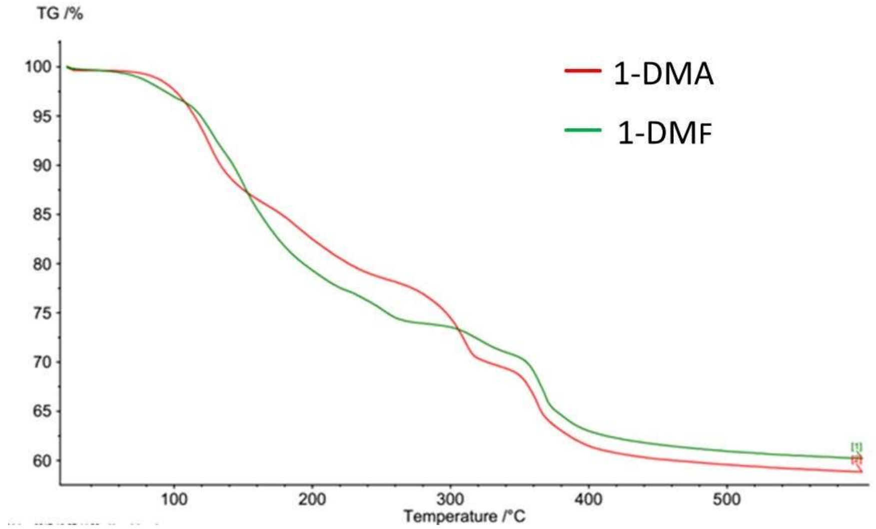

Figure S4. **TGA diagrams of 1'-MeOH-air (black) and 1'-CH_2Cl_2-air** (red)**.**

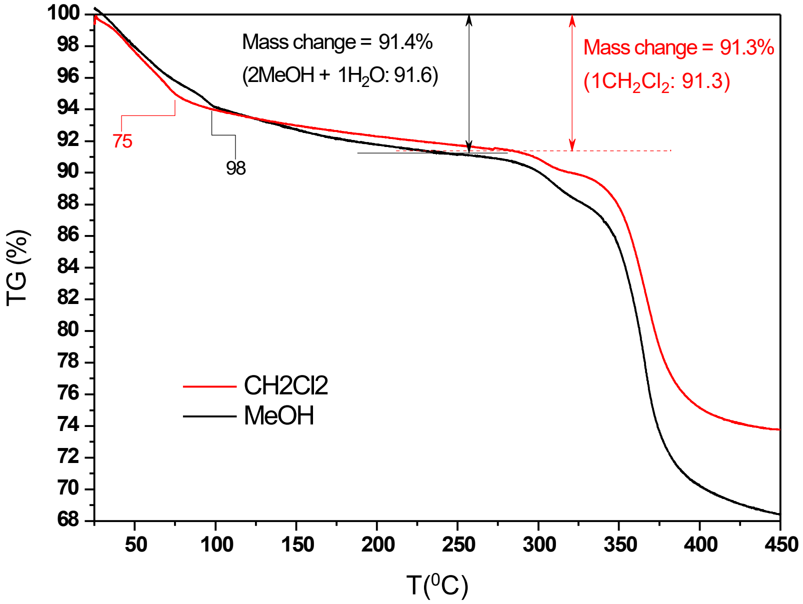

Figure S5. **PXRD comparison for MeOH related compounds.** (**A**) Calculated PXRD from structure **1-MeOH** (Figure 3). (**B**) PXRD of crystals obtained after exposing **1-MeOH** to air and dried on a filter paper. (**C**) PXRD of crystals obtained after immersing **1-DMF** into MeOH and then exposed to air and dried on a filter paper. The comparison reveals that **1-MeOH** is not stable out of solvent and rapidly transforms into **1'-MeOH-air**.

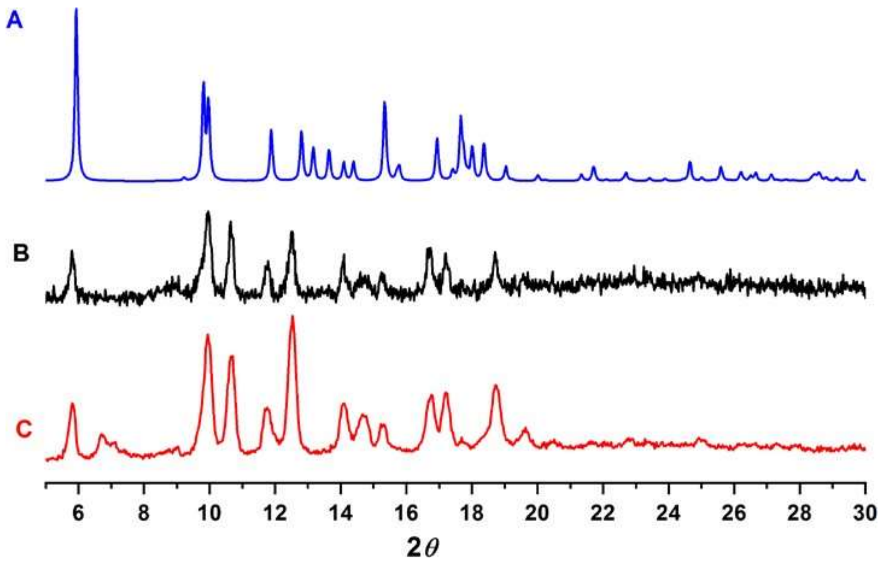

Figure S6. PXRD patterns showing reversible transformation from different samples to 1-DMF. Samples for **1'-MeOH-air**, **1'-CH_2Cl_2-air** or activated **1'-CH_2Cl_2-air** were immersed in DMF, as explained in the experimental section. The PXRD show the conversion in all cases.

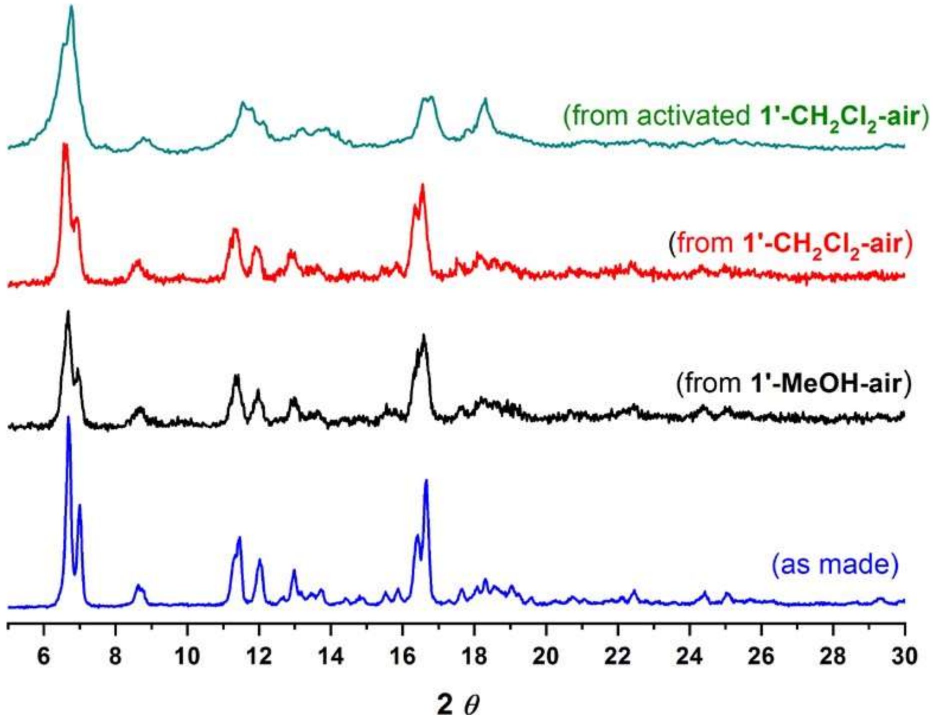

Sorption Data

Figure S7. CO_2 adsorption isotherm at 273 K for activated 1'-activated.

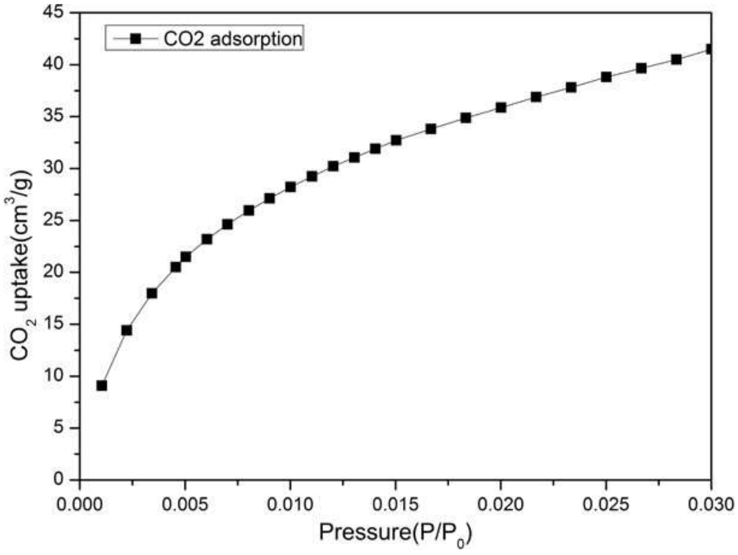

Figure S8. Pore Distribution by Density Functional Theory Model: CO2-DFT Slit Geometry for activated 1'-activated.

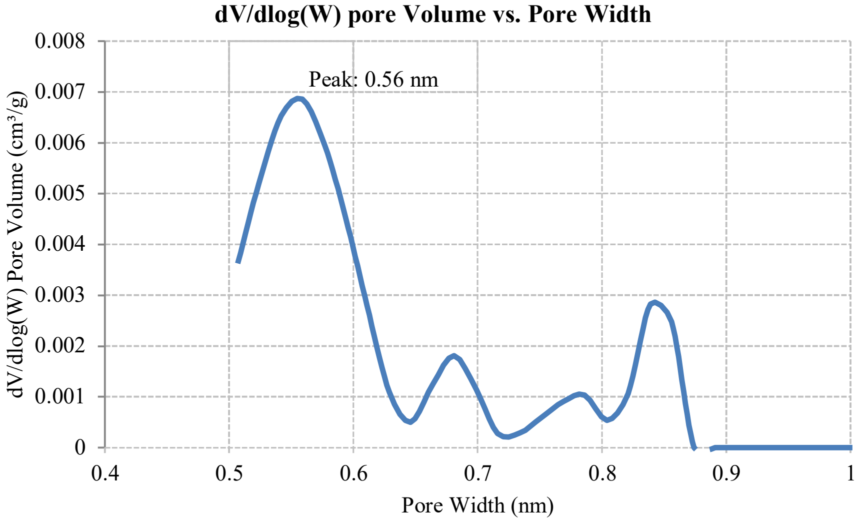

Raman Spectra

Figure S9. Comparison of Experimental Raman Spectra of 1-DMF, 1'-DCM-wet, 1'-DCM-air and 1'-MeOH-air. The Raman spectra for all compounds show that resonances for the ligand region (400-1700 cm^{-1}) are nearly identical, even for the one measured in liquid phase (**1'-CH$_2$Cl$_2$-wet**). The resonances for CH_2Cl_2 are marked with red asterisks. The characteristic double band of ν_{sym}(COO-) vibration for the bridged carboxylates at around 1400 and 1420 cm^{-1} remains unchanged, demonstrating that there is no change in the carboxylates coordination during the transformation. Major changes in the spectra are only observed in the Cu-Cu region (140-230 cm-1; bottom left zoomed area) and in the B-H region (2500-2700 cm^{-1}; bottom right zoomed area).

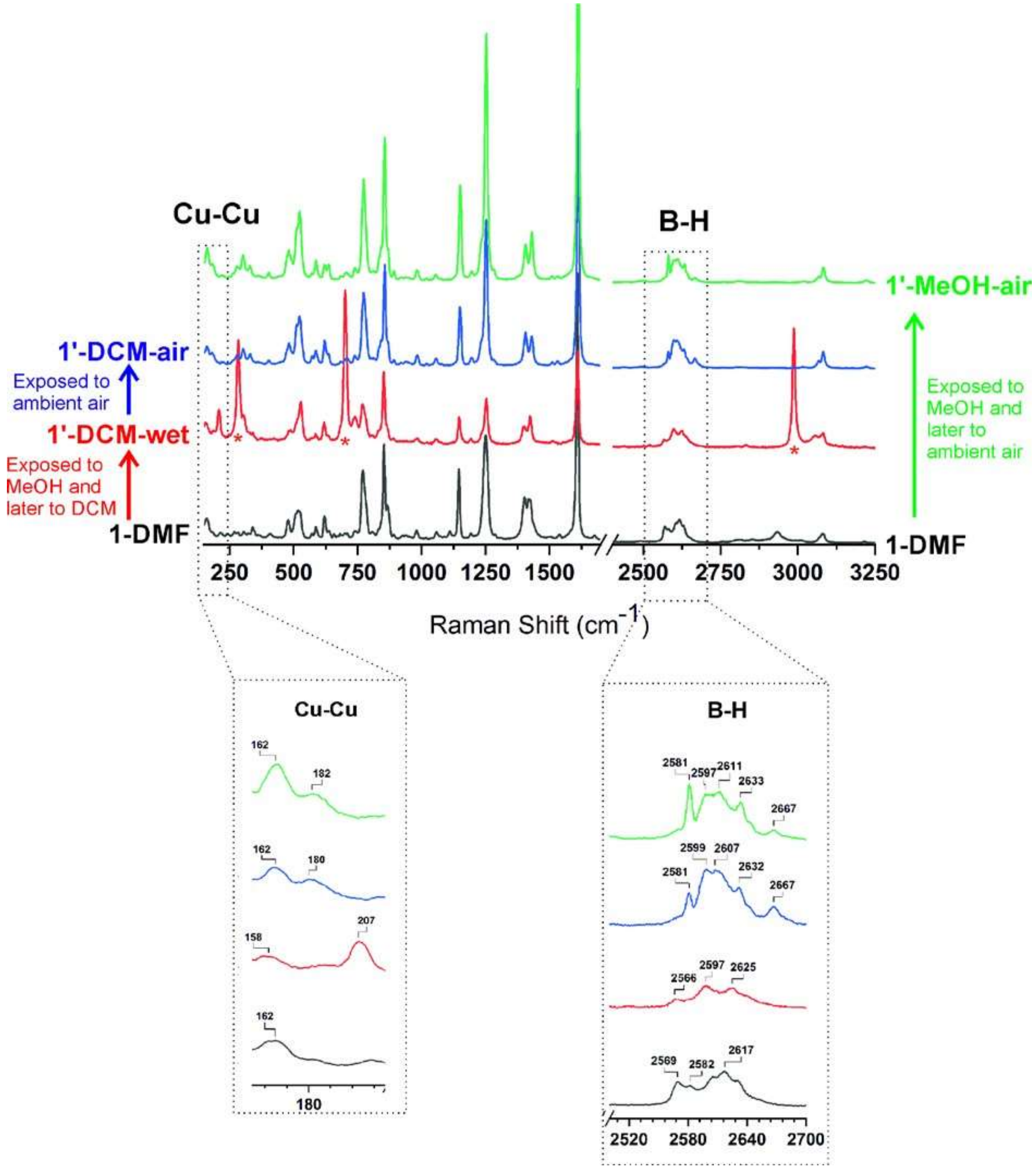

Figure S10. Comparison of Experimental and Calculated Raman for 1-DMF with [$Cu_2(L_{CB})_4(DMF)_2$] (L_{CB} = 1-(4-carboxyphenyl)-1,7-dicarba-*closo*-dodecaborane). Note the remarkable agreement between the calculated and experimental Raman spectrum for **1-DMF**. Nevertheless, as expected, predicted vibrational frequencies do not match exactly the experimental values.[1] Generally, an empirical scaling factor is applied to correct the shift and bring the values closer to the experimental ones. The figure shows the theoretically obtained spectra instead of the corrected one because our purpose here is the interpretation an assignment of the vibrational modes, not to match experimental values, as the model and the **1-DMF** are essentially different. At any rate, the agreement is remarkable.

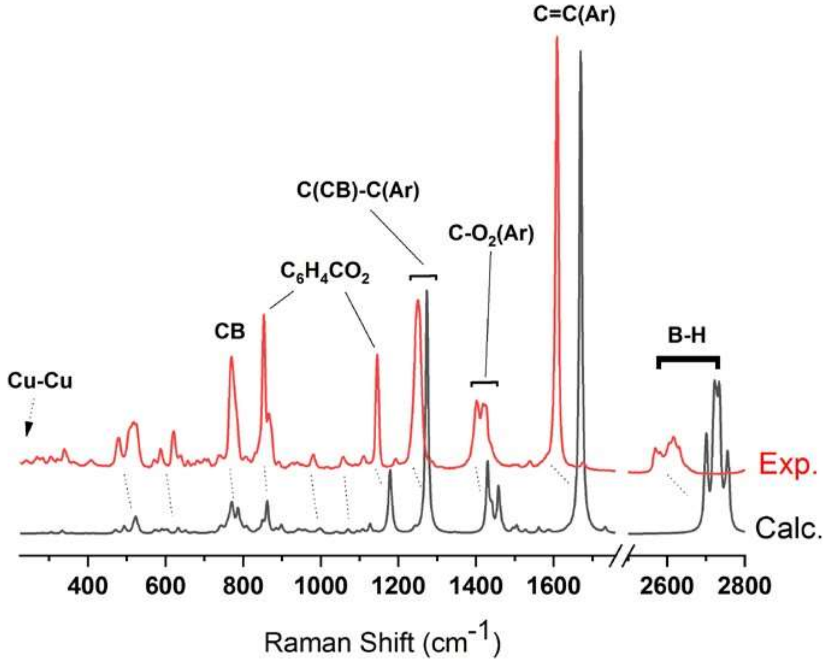

Figure S11. Comparison of Experimental and Calculated Raman for 1'-CH_2Cl_2-air and 1'-MeOH-air with [$Cu_2(L_{CB})_4(Solv)_2$] (L_{CB} = 1-(4-carboxyphenyl)-1,7-dicarba-*closo*-dodecaborane; Solv = H_2O, *m*-$C_2B_{10}H_{12}$ and/or none (Open Metal Site)). Note that calculated **1'(A)(B)** Raman spectra at the bottom correspond to [$Cu_2(L_{CB})_4$**(A)(B)**], being **A:** *m*-$C_2B_{10}H_{12}$ or CB and **B**: H_2O (violet), None (blue) or CB (green).

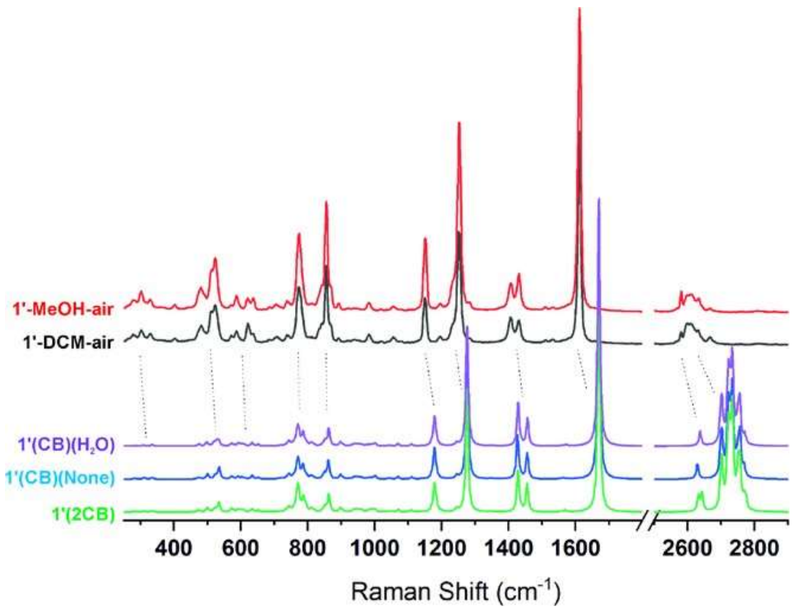

Computational details.

****
CuBiphenyl
****

98
scf done: -3000.053667

Cu	0.000122	0.000037	1.249532
O	-1.302016	-1.463159	-1.128126
O	-1.463620	-1.301676	1.125822
C	-4.845459	-4.844056	-0.001525
C	-4.389015	-4.278965	1.203113
H	-4.830821	-4.597144	2.142913
C	-3.405753	-3.295986	1.205978
H	-3.064347	-2.857257	2.137106
C	-2.850599	-2.849680	-0.001289
C	-3.296906	-3.404574	-1.208671
H	-2.857974	-3.063183	-2.139710
C	-4.280125	-4.387596	-1.206038
H	-4.598314	-4.829204	-2.145929
C	-1.796607	-1.795993	-0.001170
Cu	0.000101	-0.000119	-1.249461
O	-1.301460	1.463540	1.128157
O	1.301722	-1.463460	1.127776
O	1.463880	1.301734	1.126273
O	1.302164	1.462955	-1.127685
O	1.463384	-1.302342	-1.126194
O	-1.463240	1.302055	-1.125776
C	1.796788	1.795944	-0.000792
C	4.845285	4.844361	-0.001622
C	4.280056	4.387595	-1.206068
C	4.388851	4.279461	1.203109
H	4.598248	4.829040	-2.146035
C	3.296945	3.404467	-1.208547
H	2.858096	3.062839	-2.139538
C	2.850647	2.849763	-0.001074
C	3.405696	3.296375	1.206128
H	3.064300	2.857795	2.137330
H	4.830579	4.597877	2.142866
C	1.796238	-1.796580	0.000872
C	2.850254	-2.850242	0.001153
C	3.405332	-3.296766	-1.206064
C	3.296702	-3.404855	1.208616
H	3.063823	-2.858245	-2.137254
C	4.388671	-4.279665	-1.203079
H	4.830410	-4.598019	-2.142852
C	4.845290	-4.844446	0.001635
C	4.280004	-4.387795	1.206105
H	4.598288	-4.829192	2.146061
H	2.857834	-3.063295	2.139622
C	-1.796065	1.796449	0.001224
C	-2.850129	2.850064	0.001391
C	-3.296471	3.404924	1.208780
C	-3.405356	3.296297	-1.205866
H	-2.857496	3.063583	2.139816
C	-4.279809	4.387829	1.206155
H	-4.598025	4.829413	2.146047
C	-4.845236	4.844192	0.001645

C	-4.388736	4.279155	-1.202992
H	-4.830588	4.597287	-2.142788
H	-3.063928	2.857586	-2.136996
C	-5.894336	-5.892653	-0.001672
C	-6.893491	-5.912146	-0.990004
C	-5.914236	-6.891994	0.986467
C	-7.879983	-6.897443	-0.989806
H	-6.910483	-5.132598	-1.746477
C	-6.899767	-7.878255	0.985899
H	-5.134811	-6.909331	1.743057
C	-7.887156	-7.885010	-0.002045
H	-8.648933	-6.888917	-1.757745
H	-6.891554	-8.647360	1.753686
H	-8.655574	-8.653251	-0.002190
C	5.894243	-5.892967	0.001870
C	6.893552	-5.912195	0.990057
C	5.914131	-6.892438	-0.986135
C	7.880153	-6.897385	0.989860
H	6.910602	-5.132509	1.746384
C	6.899770	-7.878589	-0.985564
H	5.134623	-6.909947	-1.742637
C	7.887293	-7.885099	0.002245
H	8.649224	-6.888649	1.757677
H	6.891543	-8.647794	-1.753252
H	8.655798	-8.653252	0.002385
C	5.894059	5.893058	-0.001942
C	5.913857	6.892579	0.986017
C	6.893212	5.912475	-0.990276
C	6.899296	7.878932	0.985280
H	5.134427	6.909978	1.742601
C	7.879609	6.897867	-0.990250
H	6.910275	5.132802	-1.746619
C	7.886690	7.885606	-0.002661
H	6.891006	8.648170	1.752933
H	8.648557	6.889280	-1.758190
H	8.655035	8.653920	-0.002939
C	-5.894226	5.892671	0.001765
C	-5.914265	6.891918	-0.986462
C	-6.893406	5.912091	0.990076
C	-6.899937	7.878036	-0.985994
H	-5.134856	6.909275	-1.743070
C	-7.880038	6.897249	0.989780
H	-6.910317	5.132586	1.746593
C	-7.887337	7.884737	0.001938
H	-6.891833	8.647064	-1.753860
H	-8.649006	6.888668	1.757701
H	-8.655866	8.652867	0.002002

****

CuBiphenyl_MeOH

****

110

scf done: -3231.544026

Cu	0.007802	0.003345	-1.295103
O	-1.654136	-1.116102	1.100329
O	-1.424517	-1.385594	-1.132303
O	-0.359359	-0.243024	-3.497738
H	-1.328674	-0.276566	-3.427309
C	-5.319095	-4.368737	-0.014350
C	-4.655884	-4.038847	-1.210009
H	-4.969392	-4.504862	-2.139829
C	-3.584408	-3.152503	-1.212463

H	-3.069790	-2.910865	-2.136128
C	-3.143532	-2.570213	-0.016834
C	-3.795589	-2.892400	1.180946
H	-3.455247	-2.433529	2.102835
C	-4.867636	-3.778431	1.179749
H	-5.381522	-3.998765	2.111048
C	-1.989643	-1.620360	-0.015278
C	0.105139	-1.535761	-3.927368
H	-0.221774	-2.315370	-3.232142
H	1.194226	-1.484562	-3.913183
H	-0.240720	-1.752820	-4.945257
Cu	-0.030201	-0.000439	1.291780
O	1.384793	-1.414672	-1.073164
O	-1.396512	1.399750	-1.110832
O	1.408861	1.377151	-1.169307
O	1.634225	1.110464	1.064241
O	0.370020	0.076313	3.483550
O	-1.130173	1.629201	1.124901
O	1.111382	-1.618135	1.164184
C	1.969306	1.616146	-0.053669
H	1.274291	-0.279767	3.453573
C	0.418423	1.435323	3.954743
C	5.278571	4.389074	-0.076097
C	4.849562	3.786989	1.120455
C	4.601170	4.060984	-1.264201
H	5.375824	4.004467	2.045568
C	3.784419	2.892890	1.129374
H	3.465462	2.423140	2.053519
C	3.117321	2.573266	-0.060507
C	3.537130	3.165437	-1.259173
H	3.011287	2.922814	-2.176167
H	4.897756	4.535104	-2.195409
H	1.054281	2.047082	3.307191
H	-0.604869	1.807759	3.898415
H	0.768692	1.472785	4.993195
C	-1.640844	1.956961	0.005447
C	-2.610957	3.093022	0.005382
C	-3.040927	3.659690	1.212533
C	-3.105701	3.601443	-1.202958
H	-2.650028	3.267858	2.145202
C	-3.949720	4.712352	1.209889
H	-4.256550	5.158401	2.151632
C	-4.453198	5.231284	0.003446
C	-4.012457	4.655794	-1.201914
H	-4.407427	5.023924	-2.144476
H	-2.775274	3.155379	-2.134677
C	1.626354	-1.957366	0.050765
C	2.600309	-3.091025	0.064557
C	3.282430	-3.452844	-1.104746
C	2.845049	-3.803160	1.246209
H	3.084356	-2.903936	-2.018904
C	4.194489	-4.502530	-1.089681
H	4.700121	-4.787146	-2.008011
C	4.448297	-5.224618	0.090349
C	3.754202	-4.855621	1.256618
H	3.952286	-5.384000	2.184835
H	2.318380	-3.517389	2.150268
C	-6.461966	-5.314182	-0.013770
C	-6.620891	-6.253230	1.019785
C	-7.413781	-5.294204	-1.047766
C	-7.694392	-7.143107	1.018653
H	-5.881523	-6.301162	1.814369
C	-8.488049	-6.183017	-1.048531

H	-7.323436	-4.557863	-1.841552
C	-8.632822	-7.111816	-0.015471
H	-7.793408	-7.867384	1.822900
H	-9.217733	-6.144484	-1.853080
H	-9.469645	-7.804961	-0.016038
C	-5.422433	6.354113	0.001655
C	-5.346371	7.369541	-0.967235
C	-6.440947	6.430066	0.967260
C	-6.257717	8.424728	-0.970868
H	-4.549936	7.343364	-1.705685
C	-7.352982	7.484807	0.963441
H	-6.532651	5.640845	1.708245
C	-7.265415	8.486882	-0.005670
H	-6.175436	9.204048	-1.723934
H	-8.138533	7.519216	1.713736
H	-7.976108	9.308857	-0.008548
C	6.413799	5.343652	-0.085001
C	7.351012	5.338173	-1.132428
C	6.580065	6.276969	0.952577
C	8.418215	6.235402	-1.142108
H	7.254735	4.607029	-1.930260
C	7.646520	7.175235	0.942600
H	5.851252	6.314239	1.757466
C	8.570506	7.158214	-0.104759
H	9.136449	6.208326	-1.957346
H	7.751061	7.895103	1.750117
H	9.401725	7.858028	-0.112463
C	5.422117	-6.343334	0.104631
C	5.168033	-7.510847	0.844751
C	6.623270	-6.262348	-0.620698
C	6.083944	-8.562041	0.859009
H	4.233117	-7.603248	1.390413
C	7.539927	-7.312894	-0.605642
H	6.849703	-5.357384	-1.177633
C	7.274181	-8.467471	0.134157
H	5.863780	-9.459684	1.430804
H	8.467008	-7.225954	-1.166136
H	7.988351	-9.286340	0.145580

****
CuBiphenyl_DMF
****

122
scf done: -3497.143798

C	-1.920178	1.709646	-0.260060
C	-3.031426	2.706057	-0.365129
C	-3.407067	3.219885	-1.612999
H	-2.869790	2.893171	-2.496776
C	-4.444949	4.141259	-1.708538
H	-4.710534	4.550851	-2.679006
C	-5.133762	4.578189	-0.562934
C	-4.744732	4.058167	0.684621
H	-5.278327	4.365402	1.579813
C	-3.710100	3.133899	0.783272
H	-3.416212	2.729306	1.745913
C	-1.751256	-1.852874	-0.130940
C	-2.792597	-2.928515	-0.164425
C	-3.455057	-3.237420	-1.359419
H	-3.186795	-2.696903	-2.260722
C	-4.433304	-4.226352	-1.384185
H	-4.923907	-4.472055	-2.321847
C	-4.773328	-4.938670	-0.220244

C	-4.097516	-4.623602	0.972303
H	-4.359655	-5.146937	1.887728
C	-3.123128	-3.631231	1.001804
H	-2.606898	-3.387119	1.924271
C	-3.990629	-0.428511	4.863427
H	-4.379222	-0.694778	3.877064
H	-4.553241	0.434239	5.242037
H	-4.152130	-1.273634	5.544612
C	-1.882811	0.260715	5.991244
H	-1.930561	-0.558016	6.719849
H	-2.348607	1.150553	6.432264
H	-0.841478	0.474951	5.750725
C	-1.924320	-0.167290	3.586458
H	-2.550675	-0.458581	2.734360
Cu	-0.119434	0.056110	1.308426
N	-2.575387	-0.113515	4.766757
O	-1.731682	1.171623	0.875963
O	-1.251930	1.475407	-1.314891
O	-1.253763	-1.569345	1.004186
O	-1.452987	-1.302878	-1.235333
O	-0.720741	0.080804	3.438340
Cu	0.044409	-0.012790	-1.333990
O	1.510008	-1.075212	1.322149
O	1.024366	1.663909	1.156546
C	1.873302	-1.640924	0.245598
C	3.064503	-2.546257	0.308744
O	1.325367	-1.500142	-0.891247
C	3.749017	-2.741987	1.514409
C	3.521221	-3.190271	-0.849233
H	3.385226	-2.246978	2.408407
C	4.871400	-3.563381	1.558595
H	5.378979	-3.726988	2.505011
C	5.341291	-4.212478	0.402896
C	4.642491	-4.011474	-0.801386
H	5.001389	-4.485984	-1.710830
H	2.986986	-3.031193	-1.779919
C	1.668697	1.902044	0.089264
C	2.676825	3.006233	0.133246
O	1.536168	1.282914	-1.014366
C	3.009970	3.611422	1.351959
C	3.301149	3.444627	-1.041626
H	2.513537	3.275199	2.255837
C	3.954844	4.631435	1.393399
H	4.187358	5.108659	2.341212
C	4.589320	5.079370	0.220877
C	4.242305	4.467880	-0.997242
H	4.735921	4.783683	-1.912099
H	3.039186	2.971799	-1.982103
C	4.040738	-1.478340	-4.224162
H	4.450034	-0.836269	-3.440223
H	4.288054	-2.519359	-3.980196
H	4.516010	-1.220967	-5.178866
N	2.600642	-1.301377	-4.304140
C	1.874393	-2.102937	-5.278290
H	2.203408	-1.857769	-6.295476
H	2.055176	-3.169755	-5.097403
H	0.809903	-1.892252	-5.175172
C	1.946790	-0.484849	-3.452071
H	2.595840	0.028745	-2.731537
O	0.720895	-0.310382	-3.445150
C	-5.815764	-5.993697	-0.249280
C	-5.658930	-7.183430	0.482477
C	-6.986606	-5.829383	-1.009333

C	-6.639092	-8.174852	0.455196
H	-4.748700	-7.340723	1.054474
C	-7.967402	-6.820154	-1.036224
H	-7.137507	-4.906151	-1.561839
C	-7.798044	-7.997541	-0.304027
H	-6.493217	-9.091212	1.021197
H	-8.868923	-6.668742	-1.624135
H	-8.561940	-8.770073	-0.325333
C	6.540514	-5.084234	0.451275
C	6.595614	-6.277442	-0.289610
C	7.652376	-4.738054	1.238397
C	7.723438	-7.096191	-0.245140
H	5.735601	-6.577039	-0.882160
C	8.780482	-5.556405	1.283041
H	7.639457	-3.807035	1.798236
C	8.821202	-6.739212	0.541265
H	7.741077	-8.019328	-0.818603
H	9.632311	-5.265268	1.892051
H	9.700090	-7.377263	0.576225
C	5.597093	6.167031	0.267484
C	5.649525	7.146119	-0.739607
C	6.525330	6.244862	1.320060
C	6.597581	8.167696	-0.695962
H	4.923041	7.118662	-1.546952
C	7.473883	7.265933	1.363844
H	6.517236	5.482782	2.094339
C	7.514342	8.232080	0.355915
H	6.614389	8.919807	-1.480427
H	8.187855	7.302177	2.182561
H	8.253217	9.028154	0.390078
C	-6.239631	5.561398	-0.667054
C	-6.394969	6.577303	0.291804
C	-7.160107	5.501560	-1.727608
C	-7.434431	7.501573	0.193606
H	-5.677828	6.657145	1.104085
C	-8.200083	6.425195	-1.825914
H	-7.072446	4.708532	-2.465084
C	-8.342036	7.429539	-0.865663
H	-7.530134	8.284855	0.941074
H	-8.905425	6.355322	-2.649907
H	-9.152088	8.149795	-0.942396

****
CuBiphenyl_CB
****

146

scf done: -3664.394958

Cu	0.173609	0.257358	-1.267291
O	1.317477	1.318267	1.393931
O	1.453048	1.671012	-0.837665
C	5.191641	4.496707	0.948769
C	4.746308	4.167747	-0.344453
H	5.275409	4.562250	-1.207326
C	3.659884	3.321065	-0.534189
H	3.323046	3.064949	-1.532678
C	2.989497	2.776549	0.569779
C	3.423389	3.094615	1.863389
H	2.895505	2.675549	2.713272
C	4.508834	3.944436	2.047658
H	4.818194	4.207491	3.055081
C	1.833469	1.856618	0.364331
Cu	-0.297163	0.169984	1.239625

O	-1.301783	1.585027	-1.282579
O	1.535951	-1.106340	-0.781235
O	-1.133729	-1.173367	-1.540140
O	-1.801861	-0.967742	0.611223
O	0.847198	-1.413269	1.352039
O	-1.418328	1.745153	0.973468
C	-1.896281	-1.454397	-0.559504
C	-5.080722	-4.281222	-1.240196
C	-4.885075	-3.702576	0.026981
C	-4.208698	-3.913016	-2.280723
H	-5.564021	-3.951397	0.837742
C	-3.857891	-2.792065	0.247757
H	-3.717497	-2.342060	1.223810
C	-2.996956	-2.432636	-0.798278
C	-3.181398	-3.000425	-2.065667
H	-2.507040	-2.723875	-2.868969
H	-4.324808	-4.367641	-3.260336
C	1.582411	-1.687200	0.348938
C	2.588531	-2.776415	0.512463
C	2.710905	-3.450495	1.734725
C	3.425696	-3.129856	-0.554493
H	2.056375	-3.175244	2.554778
C	3.652747	-4.462841	1.883936
H	3.720027	-4.997318	2.827155
C	4.499131	-4.827665	0.821321
C	4.367453	-4.140916	-0.399335
H	5.030596	-4.386714	-1.223926
H	3.334673	-2.596840	-1.494045
C	-1.761962	2.083062	-0.206797
C	-2.798942	3.148337	-0.329321
C	-3.181712	3.623146	-1.590816
C	-3.407059	3.676375	0.817798
H	-2.701703	3.216044	-2.474158
C	-4.155685	4.609607	-1.700945
H	-4.423803	4.990358	-2.682226
C	-4.775426	5.146840	-0.558200
C	-4.381909	4.661294	0.702073
H	-4.866789	5.043108	1.595981
H	-3.112163	3.298141	1.790765
H	-0.449433	0.339170	3.353859
B	-1.302506	-0.157672	4.021883
B	-1.979795	-1.762968	3.673238
B	-0.942918	-1.512644	5.105647
B	-2.986564	-0.309403	3.472989
B	-2.562538	0.828591	4.783134
B	-1.294428	0.091328	5.793487
C	-2.423428	-2.364838	5.218672
B	-3.657329	-1.758993	4.225639
H	-1.655178	-2.512913	2.820817
B	-1.986622	-1.368251	6.546356
H	0.078668	-2.108779	5.184124
C	-3.897905	-0.232695	4.924922
H	-3.452703	0.085791	2.462766
B	-2.988869	0.080122	6.346784
H	-2.754306	1.986881	4.619435
H	-0.465051	0.740403	6.342401
B	-3.662920	-1.518723	6.002275
H	-4.528842	-2.423093	3.778973
H	-1.674421	-1.868255	7.574832
H	-4.896860	0.185079	4.865493
H	-3.469105	0.725230	7.217629
H	-4.535705	-2.039261	6.607500
H	0.496971	0.623441	-3.342330

B	1.452062	0.324958	-3.990768
B	2.143799	-1.310849	-3.961320
B	1.285556	-0.719185	-5.412554
B	3.062437	0.051781	-3.280892
B	2.760444	1.475848	-4.314989
B	1.659083	1.007865	-5.632290
C	2.799248	-1.508381	-5.536803
B	3.875760	-1.161556	-4.273813
H	1.742666	-2.252457	-3.369567
B	2.494754	-0.214528	-6.624075
H	0.305570	-1.282737	-5.769389
C	4.142907	0.491862	-4.538139
H	3.387575	0.188937	-2.155693
B	3.407430	1.144213	-5.944813
H	2.886184	2.559603	-3.852995
H	0.880862	1.768118	-6.108534
B	4.093993	-0.485556	-5.920463
H	4.708372	-1.911412	-3.893954
H	2.332073	-0.445203	-7.775380
H	5.109311	0.888104	-4.246472
H	3.967919	1.988667	-6.559573
H	5.054789	-0.834415	-6.515524
H	-2.389903	-3.438900	5.364350
H	2.825218	-2.512209	-5.946293
C	6.349643	5.402101	1.147370
C	7.286217	5.163160	2.167827
C	6.538684	6.521537	0.318625
C	8.375126	6.014234	2.352714
H	7.171714	4.288598	2.802136
C	7.626966	7.373173	0.503812
H	5.810747	6.738840	-0.458007
C	8.550373	7.123031	1.521534
H	9.092247	5.806222	3.142452
H	7.748972	8.238479	-0.142383
H	9.398722	7.786482	1.666013
C	-5.817080	6.195816	-0.679268
C	-5.879872	7.260112	0.236637
C	-6.768013	6.150171	-1.713371
C	-6.860055	8.245216	0.122275
H	-5.137248	7.327892	1.026747
C	-7.748769	7.134579	-1.827466
H	-6.752652	5.321607	-2.415937
C	-7.799131	8.186610	-0.910076
H	-6.884799	9.064641	0.835818
H	-8.479732	7.075288	-2.629589
H	-8.563031	8.954275	-0.999042
C	-6.177731	-5.251883	-1.473409
C	-6.908919	-5.238659	-2.673719
C	-6.513482	-6.208373	-0.499788
C	-7.940716	-6.150512	-2.892835
H	-6.682350	-4.489876	-3.427549
C	-7.544648	-7.120940	-0.718917
H	-5.942481	-6.252468	0.423499
C	-8.263051	-7.095932	-1.916519
H	-8.499334	-6.116863	-3.824492
H	-7.781770	-7.858208	0.043611
H	-9.066971	-7.806710	-2.087439
C	5.503908	-5.906534	0.983962
C	5.761442	-6.814711	-0.057389
C	6.222960	-6.047252	2.183459
C	6.705573	-7.829297	0.095447
H	5.196550	-6.739076	-0.982360
C	7.167788	-7.061165	2.336310

H	6.057638	-5.338972	2.990514
C	7.413043	-7.956903	1.293011
H	6.882709	-8.526654	-0.719135
H	7.719139	-7.146505	3.268995
H	8.148875	-8.747524	1.412196

****

CuPhCB

****

146

scf done: -3399.630102

Cu	0.133935	-0.126625	-1.258108
O	-1.199780	1.323296	1.123276
O	-1.362297	1.141344	-1.128639
C	-8.406719	6.232373	-0.993178
C	-6.096634	5.450784	0.020639
C	-4.941078	4.474885	-0.000356
C	-4.473469	3.924518	-1.202315
H	-4.928709	4.204426	-2.144104
C	-3.424961	3.009848	-1.205633
H	-3.070975	2.583524	-2.137575
C	-2.819558	2.625557	-0.003679
C	-3.275251	3.177439	1.199685
H	-2.805036	2.881027	2.130557
C	-4.323481	4.091621	1.199657
H	-4.666313	4.504996	2.140527
C	-1.711489	1.625819	-0.003354
B	-7.834155	6.973107	1.599682
H	-8.193464	7.317973	2.678062
B	-7.242543	5.320264	1.315129
H	-7.094454	4.463961	2.119828
B	-8.787987	5.829676	0.624892
H	-9.850713	5.371692	0.881546
B	-8.620925	7.528582	0.105927
H	-9.591316	8.203835	0.018699
B	-6.968591	8.071614	0.476454
H	-6.713276	9.196849	0.758468
B	-6.121585	6.701785	1.218622
H	-5.213552	6.773765	1.976157
B	-7.661025	4.859590	-0.336159
H	-7.854964	3.757457	-0.717182
B	-7.391728	7.603759	-1.184279
H	-7.536668	8.309746	-2.125181
B	-5.841318	7.090354	-0.496351
H	-4.764488	7.396175	-0.883959
B	-6.804668	5.962302	-1.451566
H	-6.520298	5.537626	-2.515753
Cu	0.135212	-0.110168	1.248970
O	-1.145134	-1.610142	-1.124357
O	1.400544	1.369555	-1.140939
O	1.625508	-1.394509	-1.127186
O	1.469922	-1.541459	1.128258
O	1.564243	1.226784	1.114006
O	-1.308801	-1.433709	1.128084
C	1.975838	-1.863265	0.004200
C	8.604917	-6.545717	1.111647
B	9.029903	-6.158492	-0.498087
B	8.813243	-7.850984	0.022310
B	7.903847	-5.157971	0.433576
B	7.556179	-7.893077	1.286391
B	7.001030	-6.237389	1.534532
C	6.334280	-5.718207	0.045495

C	5.189049	-4.730313	0.043577
B	7.512483	-5.617731	-1.223221
B	6.357352	-6.971829	-1.148543
B	6.033216	-7.348278	0.562794
C	4.611302	-4.285469	1.241307
C	4.681181	-4.242476	-1.169764
H	4.973372	-4.656406	2.192018
C	3.568187	-3.365055	1.229186
H	3.127665	-3.021851	2.158508
C	3.078280	-2.869283	0.015423
C	3.639932	-3.320076	-1.185204
H	3.256268	-2.940442	-2.125556
H	5.102078	-4.582924	-2.108223
B	8.071895	-7.284507	-1.490023
H	8.447251	-7.640915	-2.559125
B	7.157733	-8.358574	-0.382068
H	7.410490	-4.762711	-2.036053
H	10.107664	-5.725235	-0.734038
H	9.766430	-8.547086	0.132738
H	6.883829	-9.479320	-0.664577
H	5.462623	-7.017330	-1.924257
H	8.115214	-4.059113	0.814696
H	7.665091	-8.598714	2.232397
H	4.941480	-7.627427	0.928377
H	6.705738	-5.797763	2.589774
C	1.873398	1.730468	-0.014515
C	2.869866	2.841565	-0.014650
C	3.361713	3.347990	1.193887
C	3.307109	3.402285	-1.220659
H	3.018101	2.917649	2.127812
C	4.273767	4.398719	1.196416
H	4.629912	4.786367	2.142388
C	4.719675	4.962806	-0.007050
C	4.220726	4.451155	-1.214851
C	5.683096	6.129200	-0.010028
H	4.533635	4.882990	-2.158226
H	2.919814	3.015612	-2.156663
B	5.305533	7.469052	-1.043066
B	5.191819	7.615760	0.728829
B	6.651627	6.381913	-1.398568
B	6.471426	6.615605	1.457481
B	7.363851	5.842545	0.148217
C	8.015300	7.131739	-0.746273
B	6.868703	8.127810	-1.544813
B	7.721606	8.684378	-0.082293
B	8.032218	7.268283	0.957190
B	5.963064	8.904897	-0.224893
H	5.462116	9.972779	-0.364619
B	6.683817	8.374002	1.327036
H	7.126454	8.577161	-2.610903
H	4.326809	7.407049	-1.707146
H	4.121788	7.650769	1.237270
H	6.698135	9.061029	2.295763
H	8.556881	9.519759	-0.181311
H	6.709942	5.618031	-2.298141
H	6.293341	6.012467	2.459459
H	9.054122	7.155621	1.547057
H	7.829067	4.756188	0.173490
C	-1.652842	-1.920137	0.002088
C	-2.754404	-2.927154	0.002981
C	-3.214571	-3.475646	-1.200158
C	-3.356829	-3.313221	1.205946
H	-2.747029	-3.177566	-2.131873

C	-4.265884	-4.386479	-1.198999
H	-4.613321	-4.796111	-2.139828
C	-4.882107	-4.769412	0.001867
C	-4.408149	-4.224487	1.203773
C	-6.044274	-5.737477	-0.017717
H	-4.861760	-4.504668	2.146267
H	-2.999701	-2.889135	2.137678
B	-5.798990	-7.379690	0.494764
B	-6.081907	-6.985078	-1.218461
B	-6.751389	-6.247338	1.455640
B	-7.193506	-5.595506	-1.308242
B	-7.603541	-5.136117	0.345640
C	-8.356821	-6.505156	1.001674
B	-7.350784	-7.884021	1.186348
B	-8.583367	-7.797299	-0.099944
B	-8.740232	-6.095805	-0.614112
B	-6.935965	-8.350777	-0.476798
H	-6.689469	-9.477104	-0.762277
B	-7.797465	-7.243552	-1.594692
H	-7.497666	-8.591190	2.126070
H	-4.723123	-7.693962	0.878284
H	-5.176734	-7.061541	-1.978963
H	-8.162601	-7.583384	-2.672705
H	-9.558204	-8.465926	-0.011277
H	-6.460752	-5.827200	2.519946
H	-7.041895	-4.738264	-2.111244
H	-9.800540	-5.629791	-0.866285
H	-7.788467	-4.033567	0.729903
H	8.959371	6.952297	-1.248996
H	-9.165749	6.062656	-1.748906
H	9.350949	-6.389985	1.883171
H	-9.112222	-6.331883	1.760238

****

CuPhCB_MeOH

****

158

scf done: -3631.122083

Cu	0.116789	-0.131246	-1.294794
O	-1.178198	1.399993	1.135461
O	-1.451311	1.109804	-1.090211
O	-0.030638	0.171667	-3.486986
H	0.078043	1.137016	-3.520250
C	-8.464962	6.244330	-0.866302
C	-6.135938	5.471000	0.111019
C	-4.979840	4.496060	0.072115
C	-4.554315	3.917455	-1.132110
H	-5.043975	4.174050	-2.063402
C	-3.505464	3.002959	-1.149172
H	-3.184783	2.554190	-2.082750
C	-2.855722	2.646715	0.037398
C	-3.271872	3.225492	1.242045
H	-2.768879	2.947740	2.161577
C	-4.321715	4.138265	1.258141
H	-4.635008	4.569457	2.201425
C	-1.743203	1.645317	0.025291
C	-1.337445	-0.170691	-3.984976
H	-2.117642	0.333186	-3.405645
H	-1.435826	-1.247817	-3.847461
H	-1.421938	0.075972	-5.049655
B	-7.854768	6.986459	1.717534
H	-8.198658	7.329837	2.801470

B	-7.260622	5.336158	1.423371
H	-7.094524	4.480309	2.224988
B	-8.819188	5.839789	0.757649
H	-9.876180	5.377674	1.030458
B	-8.667366	7.539235	0.236362
H	-9.641759	8.210650	0.164424
B	-7.011713	8.089028	0.581396
H	-6.756697	9.215272	0.859896
B	-6.147327	6.722470	1.309218
H	-5.228859	6.801069	2.053184
B	-7.703569	4.874760	-0.221300
H	-7.899726	3.772089	-0.599809
B	-7.458277	7.619833	-1.072346
H	-7.620849	8.325311	-2.010892
B	-5.895413	7.112301	-0.408971
H	-4.826335	7.423381	-0.813595
B	-6.868862	5.980801	-1.349310
H	-6.598910	5.558832	-2.418419
Cu	0.120250	-0.074490	1.301031
O	-1.117878	-1.664583	-1.092399
O	1.320267	1.445334	-1.155736
O	1.669583	-1.338838	-1.107978
O	1.424969	-1.580889	1.126837
O	0.224759	-0.385507	3.498157
O	1.607601	1.220756	1.077644
O	-1.367684	-1.390841	1.140725
C	1.975081	-1.850908	0.012200
H	0.004396	-1.331684	3.531450
C	1.567709	-0.202188	3.983655
C	8.631017	-6.512882	1.112593
B	9.060667	-6.122225	-0.494419
B	8.842008	-7.815863	0.021062
B	7.931827	-5.123039	0.435363
B	7.581327	-7.860045	1.281256
B	7.026028	-6.204340	1.530948
C	6.362751	-5.682394	0.041476
C	5.212912	-4.699842	0.041485
B	7.545954	-5.579378	-1.222947
B	6.389494	-6.932524	-1.155729
B	6.061240	-7.313319	0.553986
C	4.587469	-4.315486	1.236178
C	4.742543	-4.162394	-1.165666
H	4.916280	-4.730285	2.181076
C	3.537647	-3.402393	1.226424
H	3.057971	-3.108695	2.153440
C	3.086532	-2.853396	0.021228
C	3.694573	-3.246955	-1.176461
H	3.339573	-2.826412	-2.110757
H	5.196970	-4.459617	-2.103219
H	2.277548	-0.787242	3.390098
H	1.789190	0.857543	3.854694
H	1.635060	-0.468165	5.044909
B	8.105244	-7.245695	-1.492075
H	8.484232	-7.599586	-2.560797
B	7.187670	-8.321983	-0.389536
H	7.451663	-4.723650	-2.035641
H	10.139303	-5.688868	-0.726464
H	9.794854	-8.512370	0.132716
H	6.914671	-9.442313	-0.674877
H	5.496754	-6.973042	-1.934102
H	8.143110	-4.025319	0.819826
H	7.687236	-8.567963	2.226035
H	4.968456	-7.593237	0.915933

H	6.728075	-5.765031	2.585759
C	1.849850	1.765160	-0.045098
C	2.843686	2.883448	-0.058748
C	3.311952	3.427502	1.142116
C	3.303003	3.411496	-1.270635
H	2.949265	3.024103	2.080902
C	4.220354	4.481655	1.132179
H	4.554803	4.900583	2.072912
C	4.690239	5.010015	-0.078320
C	4.215517	4.461660	-1.279439
C	5.651042	6.178352	-0.095410
H	4.546938	4.865162	-2.229109
H	2.935912	2.994074	-2.201602
B	5.266325	7.509386	-1.137177
B	5.157022	7.668484	0.633778
B	6.615768	6.425067	-1.488034
B	6.441836	6.677680	1.366074
B	7.333604	5.899103	0.060089
C	7.978285	7.184553	-0.844691
B	6.825798	8.170654	-1.647269
B	7.680647	8.740591	-0.190940
B	7.998990	7.332900	0.857531
B	5.921091	8.953685	-0.330588
H	5.416149	10.018974	-0.476234
B	6.647619	8.436075	1.223203
H	7.079410	8.613492	-2.717157
H	4.286348	7.437902	-1.798614
H	4.088196	7.703887	1.144712
H	6.661971	9.130179	2.186957
H	8.512655	9.578308	-0.298108
H	6.676842	5.656737	-2.383530
H	6.268418	6.079576	2.372014
H	9.022879	7.228188	1.445546
H	7.803542	4.814933	0.091594
C	-1.662695	-1.925715	0.026308
C	-2.759933	-2.943722	0.025244
C	-3.064036	-3.656767	-1.140025
C	-3.509952	-3.178688	1.182904
H	-2.478819	-3.479288	-2.035378
C	-4.103813	-4.580716	-1.148715
H	-4.320741	-5.126554	-2.059114
C	-4.872189	-4.806446	0.003164
C	-4.555355	-4.097687	1.170591
C	-6.016093	-5.795494	-0.031663
H	-5.125155	-4.258622	2.077495
H	-3.272716	-2.627886	2.086346
B	-5.863743	-7.293036	0.833691
B	-5.828546	-7.253559	-0.946531
B	-6.980482	-6.032746	1.362963
B	-6.912261	-5.955508	-1.507341
B	-7.618680	-5.196170	-0.080688
C	-8.473570	-6.440033	0.691409
B	-7.512665	-7.714212	1.324306
B	-8.490134	-7.931304	-0.151642
B	-8.556810	-6.371968	-1.015081
B	-6.798958	-8.481452	-0.110936
H	-6.500889	-9.631302	-0.115694
B	-7.445993	-7.650828	-1.562799
H	-7.826228	-8.229246	2.344759
H	-4.874995	-7.481754	1.458384
H	-4.797996	-7.435147	-1.502380
H	-7.607451	-8.209045	-2.598828
H	-9.462407	-8.608044	-0.105965

H	-6.891217	-5.397847	2.354412
H	-6.626703	-5.267563	-2.427865
H	-9.554965	-6.007922	-1.540786
H	-7.875356	-4.048156	0.035873
H	8.921615	7.004994	-1.348739
H	-9.235141	6.071906	-1.610029
H	9.374709	-6.359147	1.886770
H	-9.355833	-6.152716	1.252823

****
CuPhCB_DMF
****

170
scf done: -3896.723886

B	6.525316	6.391309	1.350708
H	6.688792	5.485279	2.091922
B	6.837512	8.087769	1.730681
H	7.287196	8.354639	2.794580
B	5.187186	7.542030	1.404822
H	4.334048	7.403913	2.213869
B	6.948221	6.106182	-0.361323
H	7.359338	5.032977	-0.638525
B	7.528177	7.634347	-1.037567
H	8.431531	7.606084	-1.804770
B	7.455394	8.864947	0.250684
H	8.327929	9.663164	0.335917
B	5.755350	9.082014	0.725974
H	5.328414	10.118355	1.119557
B	4.780570	7.974966	-0.272202
H	3.643234	8.108437	-0.577445
B	5.878526	7.093035	-1.358599
H	5.506883	6.674527	-2.400653
B	6.182960	8.802724	-0.989611
H	6.063194	9.637781	-1.825864
B	7.119092	-5.845360	1.776045
H	6.521822	-5.728512	2.788372
B	6.567154	-6.750719	0.366336
H	5.482210	-7.225943	0.379974
B	7.973267	-7.312813	1.290226
H	7.992704	-8.228711	2.042239
B	8.058879	-4.434609	1.207605
H	8.017037	-3.458211	1.872178
B	8.091079	-4.468315	-0.557463
H	8.003038	-3.445834	-1.150122
B	7.182435	-5.908858	-1.074573
H	6.516349	-5.866460	-2.052165
B	8.011164	-7.359129	-0.484744
H	7.982735	-8.385561	-1.081898
B	9.443681	-6.812182	0.413299
H	10.450510	-7.414423	0.584343
B	9.494977	-5.029871	0.367026
H	10.515677	-4.444443	0.510754
B	8.955136	-5.943286	-1.057398
H	9.594307	-5.969071	-2.058484
C	7.792331	7.222466	0.600169
C	1.625008	1.882580	0.083139
C	2.579113	3.036892	0.092366
C	2.999964	3.598880	1.302709
H	2.623620	3.187898	2.233117
C	3.889457	4.668925	1.308266
H	4.207017	5.086648	2.256632
C	4.369002	5.211028	0.105755

C	3.939775	4.645169	-1.102958
H	4.290680	5.046486	-2.045151
C	3.059035	3.567268	-1.109563
H	2.726862	3.133573	-2.046544
C	5.331786	6.378822	0.126225
C	1.944774	-1.662777	0.273937
C	3.156671	-2.544498	0.305318
C	3.871001	-2.740543	1.491436
H	3.525924	-2.258106	2.399427
C	5.011926	-3.539076	1.500402
H	5.555934	-3.670640	2.428675
C	5.461608	-4.163615	0.327364
C	4.739906	-3.962468	-0.857775
H	5.069201	-4.430408	-1.776932
C	3.602034	-3.162996	-0.868731
H	3.044125	-3.010263	-1.786086
C	6.709620	-5.019297	0.335621
C	8.808064	-5.870119	1.694878
C	4.048562	-1.457822	-4.253475
H	4.449248	-0.784928	-3.491222
H	4.481580	-1.191802	-5.225432
H	4.352942	-2.482206	-4.004621
C	1.885530	-2.203651	-5.234736
H	2.125008	-3.256838	-5.044264
H	2.174196	-1.957405	-6.263574
H	0.815137	-2.044183	-5.105155
C	1.932258	-0.542791	-3.446194
H	2.575766	0.015956	-2.754486
Cu	0.039297	-0.103045	-1.313632
N	2.598904	-1.352664	-4.292033
O	1.473602	1.269569	-1.019688
O	1.044138	1.608787	1.177399
O	1.378621	-1.524856	-0.853429
O	1.587013	-1.116902	1.361101
O	0.699609	-0.419007	-3.411979
Cu	-0.065227	-0.015227	1.351337
O	-1.407375	-1.447760	-1.122931
O	-1.318283	1.328668	-1.363571
B	-6.729490	-6.371700	-1.209096
H	-6.913570	-5.475020	-1.956029
B	-7.051878	-8.068280	-1.576972
B	-5.394742	-7.525383	-1.276553
B	-7.127543	-6.071126	0.504988
C	-7.988730	-7.190555	-0.438843
C	-5.516841	-6.359577	-0.000725
H	-7.517244	-8.338236	-2.633270
B	-7.655745	-8.832793	-0.084645
B	-5.962641	-9.061232	-0.580238
H	-4.549358	-7.378256	-2.093005
B	-4.973369	-7.953819	0.400214
H	-7.530378	-4.994316	0.780384
B	-7.707947	-7.593614	1.196679
B	-6.052880	-7.057053	1.495004
H	-8.601591	-7.556734	1.974781
B	-6.368982	-8.768916	1.140392
H	-8.533737	-9.626527	-0.154277
H	-5.546268	-10.103048	-0.970631
H	-3.832813	-8.090130	0.692087
H	-5.671257	-6.623694	2.527610
H	-6.243012	-9.600482	1.979200
B	-6.933341	5.959162	-2.008841
H	-6.447000	5.711746	-3.055729
B	-6.201802	6.935654	-0.733976

B	-7.627185	7.529516	-1.602886
B	-7.926140	4.683581	-1.248166
C	-6.474301	5.231382	-0.527870
C	-8.602341	6.131866	-1.810966
H	-5.087771	7.314837	-0.868765
B	-6.763956	6.277550	0.821101
B	-7.522917	7.735137	0.158146
H	-7.633995	8.373531	-2.435051
B	-9.059450	7.235779	-0.582449
H	-8.005097	3.652739	-1.821572
B	-7.824532	4.882757	0.501136
B	-9.245445	5.473796	-0.369473
H	-7.783860	3.912170	1.179220
B	-8.525688	6.460727	0.925396
H	-6.009405	6.246277	1.733940
H	-7.369255	8.805122	0.650719
H	-10.026813	7.903201	-0.738603
H	-10.316847	4.966631	-0.381495
H	-9.083371	6.625835	1.961358
C	-1.665535	-1.984837	-0.003674
C	-2.653384	-3.112383	-0.002535
O	-1.172626	-1.655349	1.121299
C	-3.312047	-3.478740	-1.181625
C	-2.925453	-3.814535	1.176654
H	-3.089735	-2.941581	-2.097064
C	-4.235485	-4.519783	-1.176087
H	-4.725721	-4.795490	-2.102604
C	-4.522181	-5.219457	0.005960
C	-3.845627	-4.858999	1.179679
H	-4.029638	-5.399580	2.099625
H	-2.400609	-3.541106	2.085564
C	-1.952696	1.564087	-0.289604
C	-3.107919	2.515500	-0.363053
O	-1.703041	1.062296	0.847956
C	-3.527778	3.038250	-1.590129
C	-3.786183	2.885477	0.804206
H	-2.999747	2.750699	-2.492771
C	-4.608926	3.914473	-1.648513
H	-4.917560	4.305755	-2.610239
C	-5.293113	4.287441	-0.483187
C	-4.865461	3.759768	0.744671
H	-5.379836	4.033336	1.658289
H	-3.453800	2.481330	1.754322
C	-4.214573	0.733605	4.287928
H	-4.495437	-0.067673	3.599815
H	-4.587698	0.487166	5.289365
H	-4.695730	1.662578	3.956072
N	-2.769010	0.886424	4.303731
C	-2.210558	1.925984	5.158035
H	-2.620872	2.903477	4.876607
H	-2.458369	1.728218	6.207605
H	-1.128257	1.935873	5.030431
C	-1.976841	0.150845	3.499697
H	-2.514525	-0.564184	2.863259
O	-0.743014	0.251135	3.443238
H	9.345448	-5.847981	2.636576
H	-8.997757	-6.915985	-0.726412
H	-9.208562	6.074299	-2.708303
H	8.799746	6.951587	0.896876

****

CuPhCB_CB

****

194
scf done: -4063.974056

Cu	0.039222	0.377892	1.267801
O	-1.432075	1.242585	-1.334103
O	-1.374377	1.679623	0.885646
C	-8.363175	6.785590	0.186080
C	-6.831180	4.820313	-0.688718
C	-5.540700	4.039255	-0.574407
C	-4.917604	3.842863	0.666315
H	-5.353696	4.256632	1.567153
C	-3.734228	3.117768	0.760878
H	-3.255759	2.966835	1.721743
C	-3.146711	2.572882	-0.386732
C	-3.758508	2.769917	-1.629749
H	-3.300545	2.348122	-2.517681
C	-4.941499	3.497003	-1.721309
H	-5.400624	3.643158	-2.691727
C	-1.889001	1.774815	-0.274656
B	-8.752697	5.919025	-2.401527
H	-9.141791	6.111779	-3.507154
B	-7.011983	5.902785	-2.031118
H	-6.113963	6.009281	-2.795912
B	-8.025682	7.222755	-1.431805
H	-7.923068	8.367600	-1.721301
B	-9.603729	6.532412	-0.967062
H	-10.558505	7.234994	-0.949979
B	-9.568423	4.781903	-1.279065
H	-10.536623	4.167325	-1.588647
B	-7.964050	4.399685	-1.930288
H	-7.706750	3.492210	-2.646795
B	-6.792180	6.502928	-0.385206
H	-5.831604	7.055701	0.026155
B	-9.343182	5.387917	0.375000
H	-10.103263	5.327643	1.282608
B	-8.324286	4.067064	-0.219839
H	-8.286341	2.962643	0.207599
B	-7.615699	5.373663	0.728769
H	-7.177017	5.276386	1.820567
Cu	0.290513	0.259899	-1.277861
O	1.385788	1.820448	1.132308
O	-1.254527	-1.101203	0.975346
O	1.478394	-0.945580	1.414599
O	1.949502	-0.711202	-0.785956
O	-0.715449	-1.424899	-1.197289
O	1.265209	1.949997	-1.126494
C	2.165386	-1.189233	0.371812
C	7.962930	-6.980946	0.004932
B	7.419770	-7.488548	1.544179
B	9.110212	-6.983782	1.278190
B	6.393512	-6.530513	0.464182
B	9.126635	-5.718853	0.021945
B	7.456154	-5.444669	-0.474245
C	6.619827	-4.895161	0.916298
C	5.468149	-3.926403	0.765830
B	6.531940	-6.093445	2.166399
B	7.670251	-4.727773	2.281398
B	8.233615	-4.319354	0.641519
C	5.118719	-3.400664	-0.486167
C	4.734364	-3.525193	1.892318
H	5.676688	-3.681992	-1.370878
C	4.057488	-2.509934	-0.612121
H	3.791983	-2.106562	-1.581672

C	3.321144	-2.124952	0.514128
C	3.670988	-2.636541	1.768854
H	3.102743	-2.332580	2.641018
H	4.995426	-3.906994	2.872081
B	8.210295	-6.370544	2.682842
H	8.463522	-6.697734	3.796292
B	9.268550	-5.273989	1.736282
H	5.561448	-6.148453	2.842446
H	7.142944	-8.626998	1.724055
H	9.964131	-7.806182	1.280794
H	10.273997	-4.821586	2.178139
H	7.451824	-3.849057	3.045580
H	5.413665	-6.916721	-0.072890
H	9.971952	-5.694288	-0.808608
H	8.377408	-3.191089	0.310352
H	7.133236	-5.210037	-1.585643
C	-1.336811	-1.724208	-0.129169
C	-2.240866	-2.912186	-0.177587
C	-2.378826	-3.648141	-1.360225
C	-2.958759	-3.299634	0.959194
H	-1.830921	-3.337319	-2.243310
C	-3.209319	-4.763820	-1.398076
H	-3.307720	-5.321406	-2.321819
C	-3.919061	-5.168726	-0.257581
C	-3.789835	-4.414538	0.917525
C	-4.810006	-6.389759	-0.310673
H	-4.339943	-4.692777	1.807991
H	-2.859553	-2.721885	1.870574
B	-6.527356	-6.176963	-0.192388
B	-5.766127	-6.662953	-1.728161
B	-5.601337	-6.923437	1.111215
B	-4.360683	-7.694908	-1.362109
B	-4.253493	-7.847465	0.391671
C	-5.710370	-8.586778	0.850152
B	-7.124770	-7.646786	0.594826
B	-6.728874	-9.034280	-0.453124
B	-4.953042	-9.158745	-0.570836
B	-7.235821	-7.488509	-1.170886
H	-8.281893	-7.349888	-1.716231
B	-5.890479	-8.426343	-1.896562
H	-8.013094	-7.706276	1.377319
H	-6.947253	-5.079459	-0.042023
H	-5.696368	-5.871746	-2.607530
H	-5.979419	-8.954199	-2.956990
H	-7.372246	-10.026262	-0.366603
H	-5.424374	-6.470107	2.186890
H	-3.355547	-7.612418	-1.982545
H	-4.413325	-10.214108	-0.565704
H	-3.254276	-7.914943	1.019844
C	1.674257	2.348250	0.012309
C	2.574741	3.540357	0.031313
C	3.030310	4.062767	1.247404
C	2.975009	4.144275	-1.166017
H	2.720282	3.593123	2.174232
C	3.871459	5.170497	1.264103
H	4.215337	5.560229	2.214719
C	4.275698	5.782235	0.068029
C	3.816265	5.252693	-1.146320
C	5.192332	6.985036	0.105106
H	4.115248	5.702023	-2.085258
H	2.621274	3.738300	-2.107244
B	6.848659	6.797681	-0.380369
B	6.416973	7.062364	1.326769

B	5.701252	7.713414	-1.358314
B	4.989493	8.126728	1.395065
B	4.544263	8.524422	-0.265837
C	5.898477	9.325489	-0.897241
B	7.313032	8.366217	-1.060582
B	7.164192	9.591958	0.226516
B	5.449368	9.690340	0.711433
B	7.766050	7.963199	0.609267
H	8.895351	7.754103	0.912708
B	6.611674	8.784523	1.709818
H	8.030362	8.538776	-1.988340
H	7.205453	5.733829	-0.760327
H	6.509669	6.157527	2.085800
H	6.921189	9.158776	2.793912
H	7.800815	10.589228	0.151085
H	5.307431	7.412069	-2.429798
H	4.124701	7.947458	2.183808
H	4.944452	10.734119	0.957953
H	3.442873	8.674295	-0.668180
H	-5.683249	-9.221252	1.729191
H	-8.484988	7.583324	0.910518
H	8.049485	-7.731027	-0.773531
H	5.712642	10.076472	-1.657212
H	0.204549	0.158858	-3.375933
B	0.845912	-0.532241	-4.106604
B	1.522414	-2.102439	-3.613368
B	0.185818	-2.026098	-4.795338
B	2.602229	-0.726492	-3.931358
B	1.927858	0.190477	-5.309125
B	0.427945	-0.607072	-5.844274
C	1.562763	-3.004855	-5.071471
B	3.019870	-2.337039	-4.521199
H	1.379198	-2.659331	-2.582934
B	0.863415	-2.229975	-6.434427
H	-0.848292	-2.538736	-4.525142
C	3.148671	-0.986585	-5.537920
H	3.310519	-0.196940	-3.147091
B	1.940889	-0.859183	-6.752279
H	2.198223	1.339132	-5.420254
H	-0.481220	0.000836	-6.306684
B	2.614362	-2.419776	-6.265192
H	3.944936	-2.980411	-4.159948
H	0.296861	-2.878894	-7.248270
H	4.148963	-0.649594	-5.786783
H	2.226197	-0.422767	-7.816505
H	3.297269	-3.112520	-6.937592
H	-0.140643	0.691731	3.367439
B	-1.055486	0.430165	4.088206
B	-1.740963	-1.204769	4.186504
B	-0.802670	-0.536539	5.552896
B	-2.702276	0.117075	3.488026
B	-2.346874	1.595396	4.426165
B	-1.169221	1.199091	5.701036
C	-2.304161	-1.318972	5.804394
B	-3.453204	-1.041648	4.589158
H	-1.367176	-2.176523	3.625979
B	-1.942991	0.030589	6.802983
H	0.198138	-1.080207	5.881127
C	-3.710029	0.623327	4.779350
H	-3.095496	0.192055	2.378745
B	-2.898181	1.350202	6.105647
H	-2.501684	2.653340	3.915982
H	-0.368284	1.984919	6.089395

B	-3.578913	-0.278507	6.206668
H	-4.302535	-1.811893	4.297422
H	-1.714183	-0.138342	7.953394
H	-4.693297	1.002381	4.523120
H	-3.425989	2.226147	6.704625
H	-4.502591	-0.595627	6.873452
H	1.453324	-4.081036	-4.994826
H	-2.302252	-2.299608	6.267419

****
CuPhCB
****

146
scf done: -3399.630102

Cu	0.133935	-0.126625	-1.258108
O	-1.199780	1.323296	1.123276
O	-1.362297	1.141344	-1.128639
C	-8.406719	6.232373	-0.993178
C	-6.096634	5.450784	0.020639
C	-4.941078	4.474885	-0.000356
C	-4.473469	3.924518	-1.202315
H	-4.928709	4.204426	-2.144104
C	-3.424961	3.009848	-1.205633
H	-3.070975	2.583524	-2.137575
C	-2.819558	2.625557	-0.003679
C	-3.275251	3.177439	1.199685
H	-2.805036	2.881027	2.130557
C	-4.323481	4.091621	1.199657
H	-4.666313	4.504996	2.140527
C	-1.711489	1.625819	-0.003354
B	-7.834155	6.973107	1.599682
H	-8.193464	7.317973	2.678062
B	-7.242543	5.320264	1.315129
H	-7.094454	4.463961	2.119828
B	-8.787987	5.829676	0.624892
H	-9.850713	5.371692	0.881546
B	-8.620925	7.528582	0.105927
H	-9.591316	8.203835	0.018699
B	-6.968591	8.071614	0.476454
H	-6.713276	9.196849	0.758468
B	-6.121585	6.701785	1.218622
H	-5.213552	6.773765	1.976157
B	-7.661025	4.859590	-0.336159
H	-7.854964	3.757457	-0.717182
B	-7.391728	7.603759	-1.184279
H	-7.536668	8.309746	-2.125181
B	-5.841318	7.090354	-0.496351
H	-4.764488	7.396175	-0.883959
B	-6.804668	5.962302	-1.451566
H	-6.520298	5.537626	-2.515753
Cu	0.135212	-0.110168	1.248970
O	-1.145134	-1.610142	-1.124357
O	1.400544	1.369555	-1.140939
O	1.625508	-1.394509	-1.127186
O	1.469922	-1.541459	1.128258
O	1.564243	1.226784	1.114006
O	-1.308801	-1.433709	1.128084
C	1.975838	-1.863265	0.004200
C	8.604917	-6.545717	1.111647
B	9.029903	-6.158492	-0.498087
B	8.813243	-7.850984	0.022310
B	7.903847	-5.157971	0.433576

B	7.556179	-7.893077	1.286391
B	7.001030	-6.237389	1.534532
C	6.334280	-5.718207	0.045495
C	5.189049	-4.730313	0.043577
B	7.512483	-5.617731	-1.223221
B	6.357352	-6.971829	-1.148543
B	6.033216	-7.348278	0.562794
C	4.611302	-4.285469	1.241307
C	4.681181	-4.242476	-1.169764
H	4.973372	-4.656406	2.192018
C	3.568187	-3.365055	1.229186
H	3.127665	-3.021851	2.158508
C	3.078280	-2.869283	0.015423
C	3.639932	-3.320076	-1.185204
H	3.256268	-2.940442	-2.125556
H	5.102078	-4.582924	-2.108223
B	8.071895	-7.284507	-1.490023
H	8.447251	-7.640915	-2.559125
B	7.157733	-8.358574	-0.382068
H	7.410490	-4.762711	-2.036053
H	10.107664	-5.725235	-0.734038
H	9.766430	-8.547086	0.132738
H	6.883829	-9.479320	-0.664577
H	5.462623	-7.017330	-1.924257
H	8.115214	-4.059113	0.814696
H	7.665091	-8.598714	2.232397
H	4.941480	-7.627427	0.928377
H	6.705738	-5.797763	2.589774
C	1.873398	1.730468	-0.014515
C	2.869866	2.841565	-0.014650
C	3.361713	3.347990	1.193887
C	3.307109	3.402285	-1.220659
H	3.018101	2.917649	2.127812
C	4.273767	4.398719	1.196416
H	4.629912	4.786367	2.142388
C	4.719675	4.962806	-0.007050
C	4.220726	4.451155	-1.214851
C	5.683096	6.129200	-0.010028
H	4.533635	4.882990	-2.158226
H	2.919814	3.015612	-2.156663
B	5.305533	7.469052	-1.043066
B	5.191819	7.615760	0.728829
B	6.651627	6.381913	-1.398568
B	6.471426	6.615605	1.457481
B	7.363851	5.842545	0.148217
C	8.015300	7.131739	-0.746273
B	6.868703	8.127810	-1.544813
B	7.721606	8.684378	-0.082293
B	8.032218	7.268283	0.957190
B	5.963064	8.904897	-0.224893
H	5.462116	9.972779	-0.364619
B	6.683817	8.374002	1.327036
H	7.126454	8.577161	-2.610903
H	4.326809	7.407049	-1.707146
H	4.121788	7.650769	1.237270
H	6.698135	9.061029	2.295763
H	8.556881	9.519759	-0.181311
H	6.709942	5.618031	-2.298141
H	6.293341	6.012467	2.459459
H	9.054122	7.155621	1.547057
H	7.829067	4.756188	0.173490
C	-1.652842	-1.920137	0.002088
C	-2.754404	-2.927154	0.002981

C	-3.214571	-3.475646	-1.200158
C	-3.356829	-3.313221	1.205946
H	-2.747029	-3.177566	-2.131873
C	-4.265884	-4.386479	-1.198999
H	-4.613321	-4.796111	-2.139828
C	-4.882107	-4.769412	0.001867
C	-4.408149	-4.224487	1.203773
C	-6.044274	-5.737477	-0.017717
H	-4.861760	-4.504668	2.146267
H	-2.999701	-2.889135	2.137678
B	-5.798990	-7.379690	0.494764
B	-6.081907	-6.985078	-1.218461
B	-6.751389	-6.247338	1.455640
B	-7.193506	-5.595506	-1.308242
B	-7.603541	-5.136117	0.345640
C	-8.356821	-6.505156	1.001674
B	-7.350784	-7.884021	1.186348
B	-8.583367	-7.797299	-0.099944
B	-8.740232	-6.095805	-0.614112
B	-6.935965	-8.350777	-0.476798
H	-6.689469	-9.477104	-0.762277
B	-7.797465	-7.243552	-1.594692
H	-7.497666	-8.591190	2.126070
H	-4.723123	-7.693962	0.878284
H	-5.176734	-7.061541	-1.978963
H	-8.162601	-7.583384	-2.672705
H	-9.558204	-8.465926	-0.011277
H	-6.460752	-5.827200	2.519946
H	-7.041895	-4.738264	-2.111244
H	-9.800540	-5.629791	-0.866285
H	-7.788467	-4.033567	0.729903
H	8.959371	6.952297	-1.248996
H	-9.165749	6.062656	-1.748906
H	9.350949	-6.389985	1.883171
H	-9.112222	-6.331883	1.760238

****
CuPhCB_1CB
****

170
scf done: -3731.802510

Cu	-0.347428	-0.187693	0.718302
O	1.021250	-1.474071	-1.757295
O	0.836285	-1.738389	0.483678
C	7.040959	-7.887275	0.182863
C	5.846865	-5.699102	-0.694510
C	4.654943	-4.768202	-0.675493
C	4.023080	-4.418620	0.526658
H	4.374659	-4.828007	1.465851
C	2.948794	-3.534270	0.534580
H	2.466298	-3.261099	1.466267
C	2.483321	-2.979258	-0.663388
C	3.107507	-3.324741	-1.867241
H	2.750256	-2.888271	-2.793290
C	4.181540	-4.208669	-1.871667
H	4.660369	-4.455764	-2.811405
C	1.359908	-1.995932	-0.647014
B	7.872749	-6.857405	-2.237231
H	8.392137	-7.001941	-3.295614
B	6.109410	-6.685212	-2.097069
H	5.326193	-6.644033	-2.984047
B	6.894310	-8.149931	-1.500459

H	6.722943	-9.248424	-1.911295
B	8.450576	-7.675060	-0.769615
H	9.320509	-8.477030	-0.695333
B	8.628528	-5.912465	-0.913876
H	9.685876	-5.384131	-1.031428
B	7.175313	-5.305028	-1.732035
H	7.097398	-4.318880	-2.384165
B	5.599799	-7.388061	-0.561677
H	4.542094	-7.865283	-0.336307
B	8.115088	-6.624922	0.631622
H	8.743041	-6.721217	1.632325
B	7.323215	-5.156922	0.036812
H	7.332089	-4.093190	0.558709
B	6.362976	-6.452606	0.754425
H	5.778920	-6.399120	1.779397
Cu	-0.451838	-0.163897	-1.816209
O	-1.925202	-1.371974	0.573431
O	1.205064	1.006522	0.386188
O	-1.531341	1.376295	0.675668
O	-1.876154	1.172315	-1.553880
O	0.881485	1.268795	-1.837190
O	-1.767514	-1.599140	-1.674438
C	-2.080466	1.716788	-0.421105
C	-6.656496	8.427389	-1.426823
B	-5.933011	9.003163	0.012057
B	-7.704116	8.851197	-0.139072
B	-5.197711	7.709302	-0.946881
B	-8.060281	7.464057	-1.201488
B	-6.518043	6.766435	-1.695492
C	-5.740604	6.238965	-0.263740
C	-4.811367	5.046248	-0.310342
B	-5.331171	7.542187	0.804843
B	-6.728234	6.487967	1.137544
B	-7.454111	5.997768	-0.412535
C	-4.510843	4.405999	-1.520949
C	-4.218264	4.568786	0.868015
H	-4.955525	4.751741	-2.445708
C	-3.635703	3.325088	-1.555999
H	-3.402715	2.836665	-2.495479
C	-3.041453	2.859081	-0.377822
C	-3.343244	3.487646	0.836225
H	-2.883451	3.125037	1.748841
H	-4.436055	5.047382	1.815277
B	-6.881414	8.247596	1.315634
H	-6.998043	8.766605	2.377775
B	-8.199263	7.293661	0.560532
H	-4.335626	7.460999	1.441314
H	-5.410286	10.067007	0.021783
H	-8.361989	9.833154	-0.230956
H	-9.251845	7.135075	1.087629
H	-6.670315	5.695477	2.016258
H	-4.188728	7.795568	-1.556900
H	-8.935514	7.514620	-1.999133
H	-7.854932	4.895156	-0.576291
H	-6.319926	6.319777	-2.770170
C	1.486327	1.515353	-0.743822
C	2.628257	2.476160	-0.788878
C	3.014520	3.063007	-1.999109
C	3.334205	2.786874	0.379447
H	2.472005	2.815102	-2.904605
C	4.084626	3.950895	-2.037891
H	4.375997	4.388481	-2.985007
C	4.790934	4.274649	-0.869902

C	4.403947	3.675466	0.337493
C	5.963305	5.228191	-0.932889
H	4.942457	3.893729	1.251674
H	3.040882	2.324557	1.314592
B	7.547346	4.608963	-0.592409
B	7.076903	5.071581	-2.247707
B	6.737679	5.722298	0.512593
B	5.966220	6.461451	-2.152902
B	5.748053	6.856793	-0.448684
C	7.302131	7.257520	0.102580
B	8.437980	5.969415	0.109384
B	8.513384	7.266626	-1.111196
B	6.850842	7.817155	-1.448453
B	8.657509	5.565629	-1.607672
H	9.674309	5.101426	-2.009476
B	7.674272	6.711344	-2.575339
H	9.238879	5.902992	0.980608
H	7.648025	3.469188	-0.285448
H	6.875975	4.218819	-3.045433
H	7.991042	7.062034	-3.665068
H	9.383912	8.068955	-1.048429
H	6.350371	5.465262	1.598244
H	5.034462	6.559632	-2.876732
H	6.609009	8.965988	-1.611713
H	4.750531	7.251082	0.048147
C	-2.284257	-1.868289	-0.540325
C	-3.412789	-2.846093	-0.519988
C	-4.011418	-3.206527	0.693074
C	-3.894599	-3.398845	-1.712127
H	-3.640073	-2.774595	1.615656
C	-5.075398	-4.102130	0.711386
H	-5.533779	-4.360805	1.658200
C	-5.563851	-4.660168	-0.479390
C	-4.959083	-4.294947	-1.690594
C	-6.743349	-5.606421	-0.444925
H	-5.324220	-4.700276	-2.626232
H	-3.432907	-3.116392	-2.651633
B	-8.237163	-5.079959	-1.152152
B	-8.060596	-5.232183	0.613759
B	-7.272004	-6.361494	-1.888607
B	-6.972615	-6.600230	0.958171
B	-6.477590	-7.291784	-0.586940
C	-7.923268	-7.806510	-1.311201
B	-9.019604	-6.556264	-1.739822
B	-9.320996	-7.614674	-0.337254
B	-7.748263	-8.072566	0.368699
B	-9.518354	-5.854823	-0.184569
H	-10.580372	-5.340194	-0.049443
B	-8.731608	-6.794472	1.124308
H	-9.661309	-6.657630	-2.731196
H	-8.266865	-4.015085	-1.670394
H	-7.984499	-4.247345	1.268102
H	-9.233130	-6.948867	2.189962
H	-10.181942	-8.427280	-0.401107
H	-6.703986	-6.297948	-2.921863
H	-6.176798	-6.552615	1.833605
H	-7.557078	-9.170186	0.773333
H	-5.417702	-7.755761	-0.829475
H	7.365478	7.985044	0.904359
H	6.981732	-8.750382	0.836759
H	-6.623955	9.067762	-2.301373
H	-7.863307	-8.666756	-1.968790
H	-0.346854	-0.439564	2.781258

B	0.537358	-0.246687	3.560218
B	1.427183	1.288011	3.622574
B	0.297669	0.846037	4.934480
B	2.255017	-0.194603	3.096268
B	1.625649	-1.544366	4.081619
B	0.410814	-0.908979	5.217111
C	1.863764	1.442367	5.276033
B	3.061853	0.932768	4.189328
H	1.239501	2.255767	2.969356
B	1.241699	0.224660	6.315103
H	-0.644754	1.536063	5.135276
C	3.074370	-0.734431	4.501242
H	2.722920	-0.398060	2.032630
B	2.064215	-1.254081	5.786652
H	1.678622	-2.646234	3.649326
H	-0.519461	-1.553613	5.575813
B	2.948356	0.276713	5.854606
H	4.029146	1.563115	3.931134
H	0.942140	0.502556	7.427343
H	4.015003	-1.255196	4.359376
H	2.416130	-2.146251	6.482786
H	3.848040	0.517316	6.583172
H	1.956150	2.445343	5.678032

****
CuPhCB_1CB_1H2O
****

173
scf done: -3808.242691

Cu	-0.343712	-0.199675	0.700979
O	1.056526	-1.483189	-1.722421
O	0.825409	-1.760423	0.511409
C	7.085111	-7.858142	0.304294
C	5.892164	-5.675432	-0.588836
C	4.694454	-4.751652	-0.586399
C	4.029190	-4.423179	0.603569
H	4.359455	-4.842882	1.545991
C	2.947630	-3.547355	0.595314
H	2.439280	-3.289940	1.517714
C	2.507966	-2.980867	-0.606838
C	3.165370	-3.305650	-1.798629
H	2.827538	-2.859253	-2.727294
C	4.246526	-4.180727	-1.787065
H	4.750147	-4.412532	-2.717728
C	1.375378	-2.006652	-0.609730
B	7.938578	-6.828587	-2.108181
H	8.469926	-6.972610	-3.160736
B	6.173365	-6.662280	-1.987027
H	5.399093	-6.624020	-2.882000
B	6.957217	-8.123561	-1.380138
H	6.794227	-9.223339	-1.791132
B	8.503922	-7.642322	-0.633414
H	9.375885	-8.441116	-0.548674
B	8.677153	-5.879308	-0.778082
H	9.733861	-5.347446	-0.885335
B	7.230664	-5.278040	-1.612100
H	7.158229	-4.293130	-2.266553
B	5.650257	-7.365020	-0.456075
H	4.591944	-7.845793	-0.241238
B	8.149702	-6.591276	0.762770
H	8.767322	-6.684201	1.770250
B	7.358942	-5.126968	0.157757

H	7.358785	-4.062586	0.678366
B	6.395737	-6.425063	0.866628
H	5.801512	-6.372182	1.885769
Cu	-0.430174	-0.181587	-1.874718
O	-1.934042	-1.373616	0.585900
O	1.220518	0.978473	0.398037
O	-1.512999	1.374508	0.712074
O	-1.897967	1.167361	-1.505479
O	0.884785	1.275656	-1.819076
O	-1.765319	-1.638982	-1.654149
C	-2.077197	1.719918	-0.374150
C	-6.587592	8.493969	-1.279704
B	-5.846653	9.052452	0.156903
B	-7.620229	8.917313	0.020176
B	-5.130746	7.759655	-0.817855
B	-7.997997	7.541227	-1.049343
B	-6.466249	6.834442	-1.561838
C	-5.680957	6.289226	-0.141224
C	-4.764544	5.087590	-0.206488
B	-5.250771	7.580479	0.933900
B	-6.654140	6.535951	1.270665
B	-7.397489	6.064005	-0.276672
C	-4.483841	4.454490	-1.425463
C	-4.164646	4.592295	0.960897
H	-4.932597	4.814192	-2.342884
C	-3.623372	3.362902	-1.477064
H	-3.406773	2.882150	-2.424197
C	-3.022622	2.876649	-0.311002
C	-3.303743	3.500116	0.910615
H	-2.838647	3.123929	1.815008
H	-4.366268	5.065505	1.914499
B	-6.790725	8.295618	1.463104
H	-6.893575	8.807532	2.530040
B	-8.123312	7.358774	0.712346
H	-4.250554	7.485170	1.560988
H	-5.314675	10.111626	0.169940
H	-8.270107	9.905658	-0.058778
H	-9.172650	7.205191	1.247276
H	-6.595285	5.736009	2.142529
H	-4.126451	7.841670	-1.436128
H	-8.879414	7.605276	-1.839178
H	-7.809126	4.966085	-0.445441
H	-6.281877	6.394601	-2.641822
C	1.497278	1.500315	-0.727431
C	2.647860	2.452526	-0.765777
C	3.051628	3.026645	-1.976319
C	3.344862	2.765734	0.407035
H	2.516879	2.774292	-2.885266
C	4.129747	3.905062	-2.010950
H	4.434849	4.332442	-2.958459
C	4.826658	4.232035	-0.838211
C	4.422233	3.645465	0.369614
C	6.006664	5.176520	-0.896408
H	4.952899	3.866391	1.287765
H	3.038584	2.312082	1.342271
B	7.585021	4.547999	-0.546196
B	7.126733	5.010272	-2.204901
B	6.776487	5.668608	0.552151
B	6.024396	6.407343	-2.118797
B	5.799524	6.807372	-0.416595
C	7.353073	7.199403	0.142400
B	8.480632	5.904062	0.157875
B	8.570859	7.198630	-1.064680

B	6.913804	7.758899	-1.412047
B	8.706830	5.495865	-1.557145
H	9.722917	5.024563	-1.952569
B	7.736220	6.645758	-2.532218
H	9.276345	5.834320	1.033617
H	7.677298	3.408260	-0.236575
H	6.925603	4.157438	-3.002415
H	8.061209	6.992451	-3.620867
H	9.446154	7.995565	-0.998699
H	6.382238	5.416375	1.636395
H	5.097081	6.509714	-2.847770
H	6.680234	8.909006	-1.578792
H	4.801929	7.209179	0.074067
C	-2.289392	-1.886984	-0.518888
C	-3.425949	-2.857940	-0.484650
C	-4.073859	-3.142725	0.723192
C	-3.865604	-3.482165	-1.657240
H	-3.731924	-2.660974	1.632360
C	-5.146748	-4.027686	0.753597
H	-5.639934	-4.229716	1.696775
C	-5.596802	-4.650834	-0.419899
C	-4.938223	-4.368036	-1.624932
C	-6.783160	-5.587875	-0.373735
H	-5.264696	-4.833059	-2.546917
H	-3.360263	-3.269294	-2.592611
B	-8.280963	-5.065458	-1.077426
B	-8.096038	-5.203808	0.688109
B	-7.321350	-6.353670	-1.808240
B	-7.005417	-6.568260	1.039281
B	-6.520149	-7.273977	-0.503215
C	-7.969498	-7.793817	-1.215145
B	-9.067861	-6.546774	-1.648213
B	-9.361674	-7.593411	-0.235222
B	-7.785411	-8.046108	0.466369
B	-9.557475	-5.832344	-0.096669
H	-10.618276	-5.315725	0.040299
B	-8.763967	-6.761300	1.216117
H	-9.714659	-6.656119	-2.635426
H	-8.313407	-4.005041	-1.604831
H	-8.021218	-4.213332	1.333754
H	-9.260112	-6.905842	2.285612
H	-10.223464	-8.405882	-0.287767
H	-6.762430	-6.298767	-2.846909
H	-6.202645	-6.508004	1.907766
H	-7.592570	-9.140524	0.878661
H	-5.461615	-7.739805	-0.748078
H	7.416631	7.928004	0.943178
H	7.021988	-8.720567	0.958722
H	-6.557006	9.140747	-2.149607
H	-7.913457	-8.659677	-1.865663
H	-0.356572	-0.467101	2.814276
B	0.526777	-0.268530	3.591051
B	1.418252	1.266209	3.635912
B	0.295194	0.837215	4.957009
B	2.243329	-0.221188	3.119659
B	1.618255	-1.560894	4.120664
B	0.409154	-0.914700	5.255693
C	1.863119	1.436326	5.286039
B	3.055577	0.916188	4.198348
H	1.228067	2.228557	2.975434
B	1.245572	0.228836	6.339271
H	-0.645675	1.529971	5.156742
C	3.069311	-0.747647	4.526186

H	2.707221	-0.434902	2.056491
B	2.065167	-1.254810	5.820894
H	1.669706	-2.667278	3.699608
H	-0.519189	-1.555792	5.626079
B	2.950144	0.276144	5.870389
H	4.021813	1.543990	3.929804
H	0.951373	0.517371	7.450371
H	4.009058	-1.270094	4.384656
H	2.420118	-2.140499	6.523930
H	3.853198	0.523405	6.592681
H	1.957647	2.443041	5.678045
O	-1.207700	-0.075096	-3.992415
H	-1.752100	0.721150	-3.860155
H	-1.835096	-0.814036	-3.899085

****

CuPhCB_2H2O

****

152

scf done: -3552.510467

Cu	-0.094253	-0.163531	1.307433
O	1.394110	-1.476976	-1.104162
O	1.133341	-1.741650	1.125406
C	7.321860	-7.949954	0.880413
C	6.147758	-5.753999	-0.003754
C	4.963912	-4.812742	0.010338
C	4.316379	-4.472547	1.206512
H	4.649450	-4.896187	2.146051
C	3.251847	-3.575956	1.207622
H	2.761675	-3.310177	2.137421
C	2.810815	-2.996989	0.012721
C	3.450849	-3.336137	-1.185304
H	3.113935	-2.882563	-2.110640
C	4.513718	-4.233128	-1.185174
H	5.002957	-4.474066	-2.121161
C	1.694562	-2.001719	0.012587
B	8.161184	-6.937083	-1.544316
H	8.678080	-7.090495	-2.602694
B	6.399637	-6.747289	-1.402986
H	5.615064	-6.700182	-2.288667
B	7.171096	-8.217435	-0.801921
H	6.988628	-9.315879	-1.208329
B	8.732483	-7.754947	-0.074069
H	9.594585	-8.565169	0.002436
B	8.927318	-5.994586	-0.225008
H	9.989558	-5.477032	-0.345812
B	7.479501	-5.376421	-1.043554
H	7.412285	-4.392120	-1.699407
B	5.885276	-7.439726	0.135625
H	4.823195	-7.906213	0.363704
B	8.408707	-6.696241	1.323536
H	9.036538	-6.795378	2.323965
B	7.630426	-5.222525	0.724281
H	7.649839	-4.157269	1.242209
B	6.658623	-6.506387	1.447655
H	6.076685	-6.443296	2.473296
Cu	-0.072261	-0.175150	-1.280728
O	-1.667807	-1.391767	1.120731
O	1.466236	1.023625	1.139787
O	-1.303720	1.387676	1.124608
O	-1.566291	1.144233	-1.107307
O	1.216721	1.303272	-1.092238

O	-1.420786	-1.657673	-1.108307
C	-1.815155	1.707387	0.007464
C	-6.338397	8.465844	-1.060848
B	-5.934681	8.880000	0.547262
B	-7.634149	8.696098	0.035995
B	-4.960303	7.737351	-0.391367
B	-7.705941	7.442554	-1.230259
B	-6.061827	6.857171	-1.487893
C	-5.548198	6.177406	-0.002669
C	-4.587515	5.009329	-0.008600
B	-5.418501	7.351820	1.267135
B	-6.794132	6.221820	1.197873
B	-7.185912	5.908957	-0.512303
C	-4.198613	4.395882	-1.207854
C	-4.074650	4.510385	1.197949
H	-4.590348	4.750177	-2.153281
C	-3.309132	3.326246	-1.202325
H	-3.009398	2.859714	-2.133837
C	-2.787608	2.843324	0.002830
C	-3.181818	3.443244	1.204635
H	-2.780036	3.067471	2.139046
H	-4.373012	4.957999	2.138366
B	-7.073222	7.941173	1.543677
H	-7.417025	8.321386	2.615207
B	-8.170017	7.049385	0.439970
H	-4.561900	7.235127	2.075992
H	-5.480344	9.949056	0.783119
H	-8.312822	9.662395	-0.068975
H	-9.294373	6.796033	0.727526
H	-6.850467	5.326079	1.971786
H	-3.859848	7.928846	-0.778180
H	-8.414430	7.566158	-2.172427
H	-7.487201	4.823268	-0.878588
H	-5.632051	6.555591	-2.545556
C	1.768185	1.557094	0.025248
C	2.869146	2.567670	0.028968
C	3.322849	3.123396	-1.172885
C	3.462483	2.963638	1.232909
H	2.862558	2.815087	-2.104958
C	4.354376	4.056569	-1.169306
H	4.694950	4.473533	-2.109444
C	4.956291	4.455958	0.033408
C	4.494366	3.897583	1.233789
C	6.088544	5.459200	0.015875
H	4.941241	4.185625	2.177329
H	3.109694	2.532178	2.163061
B	7.703893	4.906359	0.331440
B	7.211464	5.419165	-1.300384
B	6.838712	5.924273	1.483297
B	6.027545	6.742011	-1.149349
B	5.791638	7.050095	0.572092
C	7.321765	7.505119	1.142669
B	8.522651	6.278922	1.094813
B	8.530931	7.632516	-0.065296
B	6.841285	8.109373	-0.382670
B	8.763587	5.964520	-0.637097
H	9.802596	5.571733	-1.057999
B	7.722241	7.100693	-1.555641
H	9.325414	6.215763	1.964604
H	7.865064	3.761031	0.587605
H	7.065315	4.594803	-2.138591
H	8.020862	7.514785	-2.628245
H	9.358852	8.475222	0.035543

H	6.471109	5.599260	2.557059
H	5.089560	6.813843	-1.868844
H	6.540649	9.250672	-0.493821
H	4.775218	7.370067	1.084010
C	-1.985952	-1.906523	0.002617
C	-3.125789	-2.876286	-0.004809
C	-3.856995	-3.118253	1.163866
C	-3.488713	-3.541109	-1.181425
H	-3.580914	-2.602781	2.076715
C	-4.933611	-3.998371	1.153514
H	-5.489882	-4.166146	2.067811
C	-5.307600	-4.660141	-0.025520
C	-4.565483	-4.423112	-1.191158
C	-6.499150	-5.591585	-0.023504
H	-4.829265	-4.921646	-2.115779
H	-2.920805	-3.361046	-2.087328
B	-7.974761	-5.064328	-0.770980
B	-7.844181	-5.209283	0.998286
B	-6.994605	-6.351132	-1.476679
B	-6.765116	-6.575193	1.379087
B	-6.234558	-7.277060	-0.150877
C	-7.661797	-7.792533	-0.907929
B	-8.745476	-6.542707	-1.369795
B	-9.082635	-7.594293	0.030337
B	-7.528783	-8.050693	0.778160
B	-9.281304	-5.833273	0.168800
H	-10.344942	-5.315730	0.276005
B	-8.528055	-6.767392	1.502127
H	-9.362078	-6.648290	-2.376438
H	-7.990022	-4.002020	-1.295201
H	-7.792748	-4.220996	1.649255
H	-9.056406	-6.914466	2.555688
H	-9.943039	-8.405717	-0.050934
H	-6.404616	-6.290672	-2.497761
H	-5.987455	-6.514190	2.270455
H	-7.349437	-9.146713	1.192181
H	-5.169176	-7.742704	-0.364800
H	7.347808	8.198620	1.976012
H	7.255171	-8.810064	1.537531
H	-6.172627	9.210202	-1.831920
H	-7.587088	-8.656501	-1.559113
O	-0.671111	-0.209270	-3.466945
H	-1.270249	0.556427	-3.416999
H	-1.260261	-0.980203	-3.385551
O	-0.157880	-0.770478	3.488042
H	-0.971028	-1.301559	3.420423
H	0.561815	-1.423300	3.426274

References

[1] Irikura, K. K.; Johnson, R. D.; Kacker, R. N., *The Journal of Physical Chemistry A* **2005,** *109*, 8430-8437.
